# Electrically controlled heat transport in graphite films via reversible ionic liquid intercalation

**DOI:** 10.1126/sciadv.adw8588

**Published:** 2025-07-25

**Authors:** Pietro Steiner, Saqeeb Adnan, M. Said Ergoktas, Julien Barrier, Xiaoxiao Yu, Vicente Orts, Gokhan Bakan, Jonathan Aze, Yury Malevich, Kaiyuan Wang, Pietro Cataldi, Mark Bissett, Sinan Balci, Sefik Suzer, Marat Khafizov, Coskun Kocabas

**Affiliations:** ^1^ Department of Materials, University of Manchester, Manchester, UK.; ^2^ National Graphene Institute, University of Manchester, Manchester, UK.; ^3^ Department of Mechanical and Aerospace Engineering, The Ohio State University, Columbus, USA.; ^4^ Department of Physics, University of Bath, Bath, UK.; ^5^ Department of Physics and Astronomy, University of Manchester, Manchester, UK.; ^6^ Department of Science and Engineering, Universitas Mercatorum, Rome, Italy.; ^7^ Department of Photonics, Izmir Institute of Technology, Izmir, Turkey.; ^8^ Department of Chemistry, Bilkent University, Ankara Turkey.; ^9^ Henry Royce Institute for Advanced Materials, University of Manchester, Manchester, UK.

## Abstract

The ability to control heat transport with electrical signals has been an outstanding challenge due to the lack of efficient electrothermal materials. Previous attempts have mainly concentrated on low–thermal conductivity materials and encountered various problems such as narrow dynamic range and modest on/off ratios. Here, using high–thermal conductivity graphite films, we demonstrate an electrothermal switch enabling electrically tunable heat flow at the device level. The device uses reversible electro-intercalation of ions to modulate the in-plane thermal conductivity of graphite film by more than 13-fold via tunable phonon scattering, enabling observable modulation of the thermal conductivity at the device level. We anticipate that our results could provide a realistic pathway for adaptive thermal transport, enabling electrically driven thermal devices that would find a broad spectrum of applications in aerospace and microelectronics.

## Teaser

Reversible ion intercalation enables electrically tunable heat flow on graphite thin films.

## INTRODUCTION

Electrical control of the thermal conductivity of materials could lead to various innovative applications (*1*–*3*), such as reconfigurable thermal management for space applications (*4*), smart heat-dissipating materials enabling steering of the heat flow in the desired directions (*5*, *6*), and electrically driven thermal circuits (*3*). These applications, however, require an active device, i.e., a thermal switch (*7*), which can be reversibly changed between an off-state (high thermal conduction) to an on-state (low thermal conduction) on-demand (*1*), mimicking the functionality of a transistor in electronics. This thermal action can be achieved by altering the lattice (*k*_p_) or electronic (*k*_e_) contribution to the overall thermal conduction (*k*_T_) (*8*). Literature includes various approaches to control these contributions. The electrostatic gating of two-dimensional (2D) materials is used to control the electronic contribution (*9*). Electrical switching of domains in ferroelectric materials (*10*) and doping induced phase-transitions in perovskites (*2*, *11*) and layered materials (*12*) have been investigated to control the lattice contribution (*13*). Very recently, Li *et al.* (*7*) showed that electrical control of atomic bond strengths for the molecular junctions enables thermal switching. As an alternative, mechanical means have been used to alter the form factor to achieve tunable thermal conduction (*14*–*16*) (see the benchmarking in table S1 to S5). Intercalation was previously reported to be an effective way to control thermal conductivity of layered materials. Issi *et al.* (*17*) characterized the thermal conductivity of highly oriented pyrolytic graphite intercalated with FeCl_3_ at high temperatures. These films were also investigated for thermoelectric applications (*18*). More recently, electrochemical intercalation of layered materials such as MoS_2_, black phosphorus, and LiCoO has been studied as a possible way to realize thermal transistor (*19*–*21*). Intercalation of ions from an electrolyte enables modulation of both in-plane and out-of-plane thermal conductivity. Using solid-state electrolytes would enable applications of these thermal switches for demanding environments (*22*, *23*) (see the benchmarking in table S1 for a detailed analysis of their switching performance). The low thermal conductivity of these materials limits the dynamic range thermal switching. Very recently, a large modulation of diffusivity of hot electrons on graphene has been observed within the short hydrodynamic time window (*24*). Recent experiments on Li-ion batteries suggest that thermal conductivity of active layers is modulated during charging and discharging cycles (*25*, *26*). Although these works demonstrate tunability of the thermal conductivity in the active layer, the overall thermal conductance of the devices stays constant due to the small contribution of the thin active layer to the overall thermal conduction of the device. The remaining challenge is to achieve a large modulation of in-plane thermal conductance in a scalable device layout. This paper introduces a practical realization of electrothermal switches using high–thermal conductivity graphite films.

## RESULTS

**Fig. 1.**
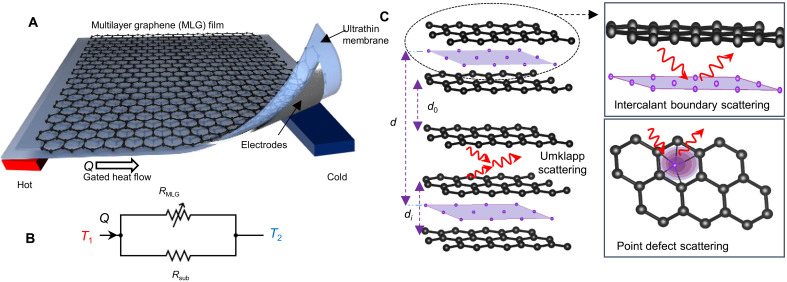
The structure and operation principle of the device. (**A**) Schematic of the thermal switch consisting of thin graphite film on a 5-μm-thick polyethylene membrane soaked with ionic liquid electrolyte and platinum counter electrode at the back. (**B**) Thermal circuit model of the device. The thermal conduction through the graphite and the substrate is represented with a variable and a fixed thermal resistor. (**C**) Representation of possible phonon scattering mechanism including Umklapp scattering, intercalation-induced point defects, and scattering from two types of boundaries.

To overcome this challenge, we have designed a device using the very high in-plane thermal conductivity (>1000 W m^−1^ K^−1^) of chemical vapor deposition (CVD)–grown graphitic film (GF) (~100 nm) on thin (~5 μm) low–thermal conductivity polymer membranes (~1.1 W m^−1^ K^−1^), soaked with an ionic liquid electrolyte [diethylmethyl(2-methoxyethyl)ammonium bis(trifluoromethylsulfonyl) imide (DEME-TFSI)]. The high thermal conductivity of graphite films primarily originates from the strong, anisotropic sp^2^ covalent bonds within graphene layers, which result in high phonon velocities and long phonon mean free paths, enabling highly efficient heat conduction (*27*). The fabrication scheme exploits lamination of the thin polymer membrane on CVD-grown GF and deposition of platinum electrodes (~50 nm) on the back of the membrane (Fig. 1A). The device uses reversible intercalation of ions to modulate the in-plane thermal conductivity of the graphite. The thickness of the film (100 nm ~ 300 layers, see figs. S1 and S2) is simultaneously optimized to achieve efficient intercalation (*28*, *29*) with large thermal conductance. The x-ray diffraction (XRD) analysis of CVD-grown graphite films shows that graphite structure is irreversibly modified after the first intercalation cycle due to the insertion of ions between the layers. Therefore, these films could also be treated as a multilayer graphene (MLG) films. Figure 1B shows the thermal circuit diagram of the device, including the two parallel thermal resistors associated with the active layer and the passive substrate. Using an ultrathin membrane reduces the thermal conductance of the substrate ( $\sigma_{\text{sub}}=\kappa_{\text{sub}}\;t_{\text{sub}}=\frac{1}{R_{\text{sub}}}$ ), which must be smaller than the thermal conductance of the active layer ( $\sigma_{\text{MLG}}=\kappa_{\text{MLG}}\;t_{\text{MLG}}=\frac{1}{R_{\text{MLG}}}$ ), such that the modulation yields an observable change in the effective conductance of the whole device ( $\sigma_{T}=\frac{1}{R_{\text{MLG}}}+\frac{1}{R_{\text{sub}}}$ ). To gain more insight for the operation of the thermal switch, we have developed an analytical circuit model for the on/off ratio of the device. The on/off ratio is given by the expression $r=\frac{R_{\text{on}}}{R_{\text{off}}}=\frac{1+\frac{R_{\text{sub}}}{R_{G,\text{on}}}}{1+\frac{R_{\text{sub}}}{R_{G,\text{off}}}}$ . When $R_{\text{sub}}\ll R_{G}$ , the heat transport is mainly dominated by the substrate leading r∼1 . To observe the modulation at the device level, $R_{\text{sub}}>R_{G}$ . We estimated that the thickness of the substrate needs to be thinner than 10 μm for observable thermal modulation. Achieving similar on/off ratio with low–thermal conductivity materials requires even thinner substrates.

The thermal conductivity of the film is controlled by the reversible intercalation of ions that can be characterized by the intercalation stage, with *n* indicating the number of graphene layers between two intercalated layers (Fig. 1C). The effective distance between periodic repeat of intercalant layer can be written as $d=\left(n-1\right)d_{0}+d_{i}$ , where *d*_0_ and *d_i_* represents van der Waals distance of graphene and intercalated layers. This electrically tunable structure enables reversible phonon scattering mainly through four mechanisms: (i) scattering from point defects due to intercalant ions, (ii) scattering from boundaries between intercalated and unintercalated layers, and (iii) Umklapp scattering leading to intrinsic reduction of conductivity. These mechanisms reduce the phonon contributions; however, (iv) the electronic contribution increases with the doping and becomes the dominant heat conduction mechanism at the intercalated state. The on/off ratio of the thermal switch is limited by the electronic contribution in the intercalated state. We first present the thermal and spectroscopic characterization of the thermal switches and then compare these results with an analytical thermal model. We have also analyzed the contribution of the out-of-plane thermal conduction on our measurements (figs. S7 and S8).

**Fig. 2.**
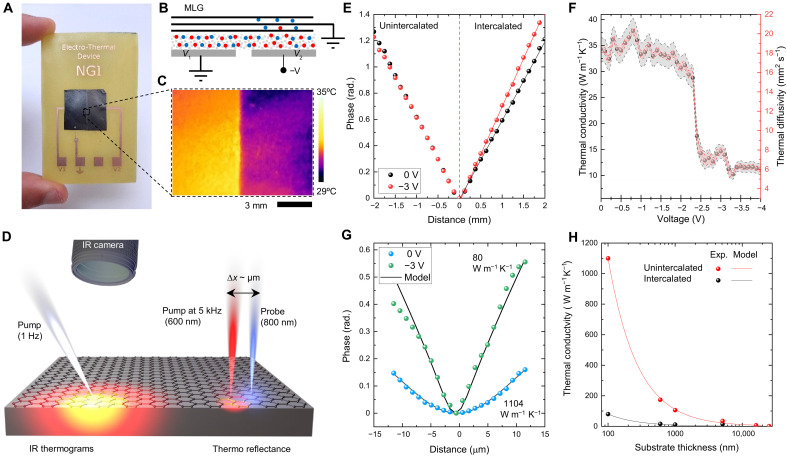
Characterization of the thermal switch. (**A**) Photograph of the fabricated device wired on a printed circuit board (PCB) board with 12-mm hole at the center with a freestanding polyethylene film that contains graphite film on the top and patterned electrodes at back. The graphite layer is wired to the ground, and back electrodes are wired to two voltage lines on the PCB. The two pattered back electrodes enabled area-selective intercalation. (**B**) Cross section and (**C**) the thermal image of the device showing that only half of the device is intercalated (low emissivity) for *V*_1_ = 0 V and *V*_2_ = −3 V. The unintercalated area (high emissivity) was used as a reference for the measurements. (**D**) Schematic showing the thermoreflectance and IR thermograms for thermal characterization in micrometer- and millimeter-length scales. For thermoreflectance measurements, the 600-nm pump laser is modulated at 5 kHz, and the thermal wave profile is measured by moving the 800-nm probe laser. IR thermography uses a camera to record the heat waves generated by a supercontinuum laser modulated at 1 Hz. (**E**) Measured phase delay of the heat waves as a function of distance from the laser spot. The change in the slope of phase for the intercalated area indicates the modulation of the thermal diffusivity *D*, which is calculated as *D* = ωr^2^/2ϕ^2^, where ω is the modulation frequency, *r* is the distance from the source, and ϕ is the phase delay. (**F**) Variation of the thermal conductivity (black) and diffusivity (red) as a function of the device voltage displaying a three-fold decrease. (**G**) Measured phase delay recorded by the thermoreflectance measurements and the fitting results of the thermoreflectance model at 0 and −3 V. (**H**) Measured (scattered plot) thermal conductivity of unintercalated and intercalated devices with different substrate thickness. Solid line shows the calculated thermal conductivity of the devices.

Figure 2A shows the fabricated device consisting of two separate back electrodes, enabling controlled intercalation on the selected area (Fig. 2B). The process can be visualized with an infrared (IR) camera due to the suppressed IR emissivity of the intercalated film (Fig. 2C) (*21*). It should be noted that the IR emissivity of the graphite film is suppressed, resulting colder appearance in thermal images. To eliminate heating-induced deintercalation, we performed the experiments using low laser power to minimize overheating and prevent thermal deintercalation. The laser was focused to a small spot, and thermal diffusivity was measured in the surrounding area using an IR macro lens, which reduces the need for large-scale heating. The suppression of the emissivity does not affect the analysis of the phase of the thermal waves. To measure the thermal conductivity of the overall device and the active layer, we implemented IR thermography (*30*) and modulated thermoreflectance (MTR) methods (Fig. 2D). These techniques measure the phase delay of thermal waves in millimeter-length (Fig. 2E) and micrometer-length (Fig. 2G) scales, providing complementary information for the device and active layer (see figs. S3 to S11). Using the continuum-based thermal heat diffusion model (*31*–*33*), we were able to extract the extrinsic and intrinsic thermal conductivity from the measured thermal wave profile in the device. This model solves the anisotropic heat diffusion equation across a series of layers in a stacked multilayer system (*31*). We compared this solution to the experimental thermal wave profiles with a least-squares minimization routine while using in-plane and out-of-plane thermal conductivity as a fitting parameter (figs. S7 and S8).

In Fig. 2F, we show the variation of actual thermal conductivity of the device with the applied voltage. Although the electronic contribution of the thermal conductivity increases by 20-fold (fig. S13), we observed a sudden decrease in the thermal conductivity after −2.5 V, indicating an enhanced phonon scattering. The reduction of in-plane thermal conductivity was further confirmed with the thermoreflectance measurements (at a modulation frequency of 1 kHz), showing that the thermal conductivity modulation of film varies from ~1100 down to 80 W m^−1^ K^−1^, providing a tunable thermal conductivity of the overall device from ~35 down to 10 W m^−1^ K^−1^ (Fig. 2G). After the first cycle, the thermal conductivity was recovered to over 95% of its initial value when the voltage was brought to +2 V to fully deintercalate the film. To avoid the effect of an additional metal layer, we have not used any metal transducer for thermoreflectance measurements (fig. S25).

The intercalation process is reversible; however, we observed that the cycling performance of the devices is limited by the residual water content in the electrolyte and oxidation of the graphene layer. Operating the devices in vacuum provides notably longer lifetime and enable to exceed 1000 switching cycles (see figs. S9 to S11). Although the thermoreflectance provides the modulation of the active layer, IR thermogram results are affected by the substrate thickness that limits the contribution of the active layer to the overall device thermal conductance. To understand the effect of the substrate on the modulation, we have fabricated devices with different substrate thickness and compare the results with the thermal model (see figs. S19 and S20). Figure 2H shows the variation of the thermal conductivity at the on-and-off state with the substrate thickness. As the substrate gets thinner, the thermal modulation of the device is approaching the intrinsic values obtained by thermoreflectance measurements. We observed a very good agreement between the experimental results and the thermal resistance model (section S8).

The enhanced phonon scattering in the intercalated film can be attributed to a boundary scattering from intercalant graphene layer interfaces and defects resulting from the graphene’s carbon bonds ionically interacting with the dangling bonds of the intercalant ions. These ionic interactions are much stronger than the weak van der Waals interaction between the graphene layers, resulting in large scattering rates due to charged impurities. The performance of the thermal switch, i.e., on/off ratio, depends on the intercalant material and the efficiency of the intercalation process. We believe that the minimum thermal conductivity at the intercalated state is limited by the electronic contribution. We have tested this assumption using similar devices with Li-intercalation, which provides higher intercalation efficiency with doping levels resulting in larger electronic contribution limiting the on/off ratio (fig. S18). Ionic liquid (IL) intercalation provides relatively less efficiency and higher scattering rates, resulting in larger thermal modulation.

**Fig. 3.**
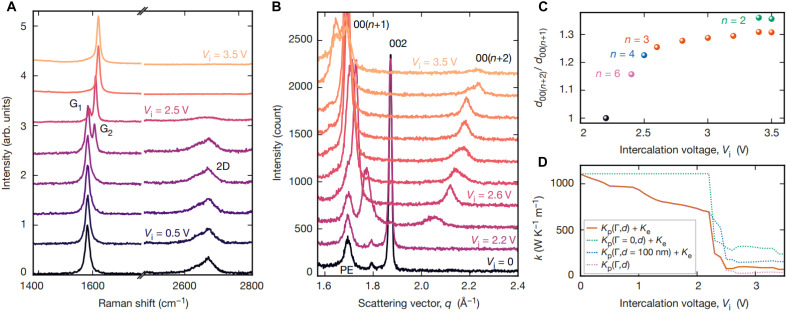
Spectroscopic characterization of the thermal switches. (**A**) In situ Raman spectrum of the graphene layer recorded at different bias voltages, showing the variation of the frequency and intensity of G and 2D bands. (**B**) Voltage-dependent in situ XRD showing the characteristic graphite peaks at 0 V and appearance of higher-order diffraction peaks 00(*n*+1) and 00(*n*+2) due to the periodic repeat of intercalant layer appears. (**C**) Ratio of peak positions 00(*n*+2)/00(*n*+1) as a function of the voltage, highlighted in different colors the dominant graphite intercalation stages, *n*. Two dominant peaks are coexisting when a voltage of 3.4 and 3.5 V is applied (fig. S9). (**D**) Predicted thermal conductivity profile of the MLG sheet as a function of bias voltage using the anisotropic thermal conductivity model. The brown line represents the model accounting for both the defect scattering strength (Γ) and the boundary scattering distance (*d*). The blue and green dotted lines displayed the cases where only one of those two effects is considered. The red dotted line is for the phonon mediated thermal conductivity without considering the electronic contribution. The model is fitted for the experimentally measured (MTR) thermal conductivity values at 0 and 2.5 V.

To elucidate the intercalation process further, we performed in situ spectroscopic characterizations. In Fig. 3A, we show the evolution of the Raman spectra of the MLG film under the bias voltage. We observed a blue shift in the frequency and splitting of the G band at higher voltages. The splitting of the G band indicates partial intercalation of the multilayer film. The G_2_ band is associated with the interfacial mode between intercalated and unintercalated layers. We also observed suppression of the 2D band due to Pauli blocking, indicating a large Fermi energy shift of >0.8 eV obtained from the condition of blocking interband transitions as $2E_{F}>E_{\text{ex}}-2\hbar \Omega_{D}$ , where *E*_F_ is the Fermi energy, $E_{\text{ex}}\;\left(1.96\;\text{eV}\right)$ is the energy of the excitation laser, and $\hbar \Omega_{D}$ (~0.17 eV) is the energy of D-band phonons (fig. S12) (*34*). However, we did not observe the Pauli blocking of the G-peak, because it requires larger Fermi energy that is not achievable with ionic liquid intercalation. It should be emphasized here that we did not observe a notable defect band (1350 cm^−1^), indicating that the intercalation and deintercalation process is not detrimental to the quality of the film. To measure the voltage-dependent intercalation stage, we performed in situ XRD on the device (Fig. 3B). When we apply voltage >2 V, the (002) peak of graphite gradually disappears and higher-order diffraction peaks due to the periodic repeat of intercalant layer appear. We estimated the intercalation stage from the ratio of the peak positions of 00(n+2)and 00(n+1) (*35*, *36*). From these results, we were able to obtain voltage-dependent intercalation stages (Fig. 3C, fig. S13, and table S8). We have supported these results with in situ x-ray photoelectron spectroscopy (XPS) providing chemically specific information for the intercalation process (fig. S14). The ionic liquid contains two nitrogen atoms, one with a positive charge (quaternized nitrogen, N^+^) and the other with a negative charge (imide nitrogen, N^−^), which yield two well-resolved N1s peaks. At 0 V, we observed symmetric N1s peaks, indicating no net charge on the layer; however, at −3.0 V, there is a notable charge imbalance ~20% (the ratio of N^−^ to N^+^), which is responsible for electrostatic doping on the layers. XPS also provides a direct measurement of the Fermi energy. Because the graphite layer is grounded, the shift in the binding energy is due to the Fermi energy change. The binding energy of N^+^ decreases by 0.7 eV at −3 V, which is consistent with p-type doping and the value obtained from Raman measurements.

On the basis of these observations, we present an analytical model to get some insight on the mechanism affecting thermal conductivity during the intercalation process. We used an empirical Klemens-Callaway model for thermal conductivity previously parameterized for graphite (*33*, *37*, *38*). The model is based on the kinetic theory of phonon gas expression obtained from the relaxation time approximation of Boltzmann transport $\kappa =\sum_{q}C{|\;\vec{v}\cdot \hat{n}\;|}^{2}\tau$ (*32*). The summation is over all the phonon modes within the Brillouin zone, *C* is specific heat, $\vec{v}$ is phonon velocity, $\hat{n}$ is direction along which thermal conductivity is calculated, and τ is phonon lifetime that considers Umklapp scattering leading to intrinsic reduction of conductivity, intercalation-induced defects acting as point defects, and scattering from two types of boundaries. In-plane mean free path of phonons is limited by the grain size and cross-plane boundary determined by the intercalation stage obtained from XRD results, that is, the number of graphene layers between successive intercalant layers. Umklapp and point defect scattering take their usual form applicable to 2D materials (*37*). The boundary scattering assuming diffuse limit is given by $\tau^{-1}=2\vec{v}\cdot {\hat{n}}_{∥}/D_{g}+2\vec{v}\cdot {\hat{n}}_{⊥}/d$ , where $D_{g}$ is grain size in-plane direction and *d* is cross-plane thickness determined by the intercalation stage, and ${\hat{n}}_{∥}$ and ${\hat{n}}_{⊥}$  are unit vectors for in-plane and cross-plane directions, respectively. For simplicity, all the phonons are described using Debye expression ω=vq , where the velocities are obtained from the solution of Christoffel equation (*32*).

To model conductivity as a function of applied voltage, we take into account the microstructural changes taking place under the external stressor (*39*). The cross-plane thickness was determined on the basis of intercalation stage obtained from XRD data. While we did not determine the absolute concentration of point defects, we assumed that it is proportional to in-plane electrical conductivity obtained from sheet resistance measurement, assuming that contribution of charged impurity defects to electrical conductivity and reduction of thermal conductivity are related. When considered, independently scattering by point defect and intercalation delamination provide qualitatively similar trends in thermal conductivity modulation obtained from thermal reflectance and IR thermogram experiments (Fig. 3D). Additionally, the electronic contribution to thermal conductivity (*k*_e_) was calculated separately using the Wiedemann-Franz law, as detailed in fig. S17, and it is not coupled to the phononic component. Sensitivity of the laterally resolved thermal wave imaging approaches to basal plane conductivity of graphene offers a notable advantage over previous reports. Considering the range of cross-plane conductivities predicted by the anisotropic thermal conductivity model, we estimate the uncertainty in measured basal plane conductivity. We obtain values of 1105 ± 50 W m^−1^ K^−1^ for in-plane assuming 6.8 ± 3.5 W m^−1^ K^−1^ for cross-plane (*8*) for pristine. Similarly, for intercalated sample, we obtain 80 ± 5 W m^−1^ K^−1^ for in-plane assuming 1.5 ± 1 W m^−1^ K^−1^ for cross-plane conductivity. For detailed explanation of the model, see section S7. The cross-plane thermal conductivity in the intercalated state was not arbitrarily chosen but estimated using an anisotropic thermal model (*40*) based on interlayer spacing and defect scattering strength, as detailed in section S8 and illustrated in figs. S15 and S16. Moreover, the extracted in-plane thermal conductivity from MTR measurements shows low sensitivity to the assumed cross-plane value, as supported by the analysis summarized in table S6 and figs. S7 and S8.

**Fig. 4.**
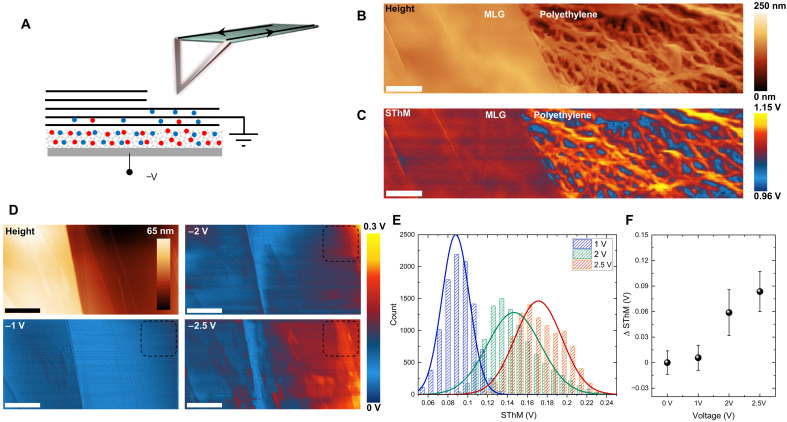
Nanoscale visualization of thermal switching. (**A**) Schematic showing the working principle of the SThM. The joule-heated AFM tip produced a local increase of temperature beneath the tip apex. Measuring the heat dissipation at different locations enabled us to estimate the thermal conductivity nature of the specimen qualitatively. (**B** and **C**) Topography and SThM maps of highly orientated pyrolytic graphite flake (HOPG) onto porous polyethylene, respectively (scale bars, 1 μm). (**D**) Height and SThM signal at different device voltages. The height map displayed a sharp step of ~25 nm. The intercalation process started from the right corner at 2 V and subsequently propagated throughout all thinner side of the samples at 2.5 V. (**E**) Histogram extracted from the dotted square (1 μm^2^) in the right side of the SThM maps showing the gradual expansion of the intercalated area. (**F**) Graph shows the shift of mean thermal voltage against the bias voltage. The measurement error was calculated by calculating the histogram’s SD.

Scanning thermal microscopy (SThM) can add additional insight to the detailed features of these devices at the nanoscopic scale (*14*, *41*). We used an atomic force microscope (AFM) tip with a resistive element near the apex to heat the sample via Joule heating and simultaneously monitored its temperature. In this experiments, we used highly orientated pyrolytic graphite flake (HOPG) exfoliated onto a porous polyethylene membrane. A change in the thermal conductivity of the sample or their temperature produces a variation in the tip resistance, providing qualitatively thermal information of the specimen (Fig. 4A). In Fig. 4 (B and C), we show the correlation between the AFM height image and thermal image of the boundary between the graphene film and the membrane. Due to the low thermal conductivity of the membrane, the thermal image shows higher voltages, indicating low thermal dissipation at a nanometer-length scale. The blue contrast signal observed between the polyethylene fibers is due to the convective cooling of the tip. To minimize the tip cooling, we performed the experiments at low scanning speed (at 0.1 Hz).

We performed in situ SThM to monitor the spatial variation of the intercalation process and the suppression of the local thermal conductivity (Fig. 4D). It is clear that high-resolution thermal contrast can be obtained in the SThM images for the intercalated regions, which gradually diffuse with the applied voltage. The area with thicker graphene layers shows slower intercalation process and thus delayed thermal switching. The SThM maps display an inhomogeneity of the intercalation process, highlighting the grain boundaries and defects that initiate the process. Figure 4 (E and F) shows the distribution of the SThM signal and its variation with the applied voltage. These SThM images show that the intercalation process is initiated from the defects at the grain boundaries on the graphene film then diffuses in the plane (fig. S21).

**Fig. 5.**
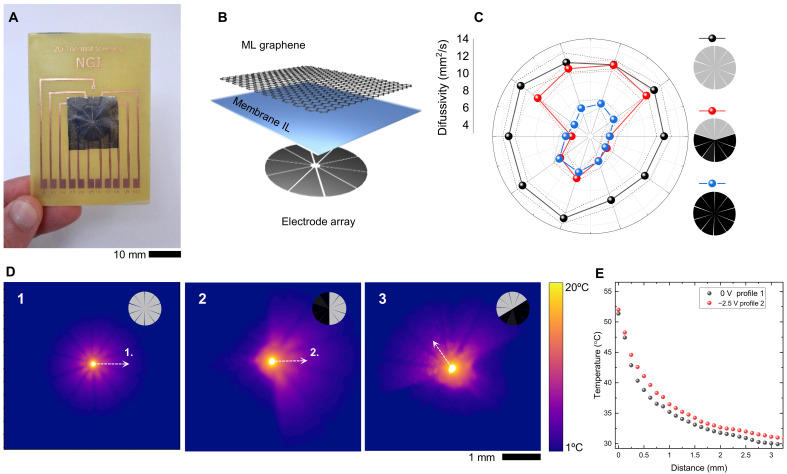
Programmable 2D steering of heat. (**A**) Optical image of the fabricated 2D thermal steering device, mounted onto a custom-built PCB. (**B**) The device structure of 2D thermal steering device: at the top, a multilayer (ML) graphene electrode, a porous PE substrate soaked with ionic liquid (IL), and 10 independent back electrodes. (**C**) In-plane radial thermal diffusivity modulation for three specific configurations: When the device is not intercalated (black dots), when over half of the device is intercalated (red dots) and when the device is fully intercalated (blue dots). (**D**) Three long-range IR thermograms are reported for different intercalation configurations, highlighting the device’s steering capability. (**E**) The modulation of thermal conduction as a function of the direction joined with the reversible intercalation process enables the preferential heat dissipation in a specific direction, producing an increase in temperature along the unintercalated region.

To showcase the promises of our approach, we demonstrate the steering of heat waves by programmable in-plane thermal diffusivity. Figure 5A shows the fabricated device and its layout consisting of a continuous graphene film with 10 independent back electrodes (Fig. 5B) shaped into equal slices. The area-selective intercalation of graphene film enables reconfigurable in-plane anisotropy, as shown in Fig. 5C for three different device configurations. To generate the heat waves, we focused the excitation laser on the center point and measured the dissipation of heat while applying different voltages on the back electrodes (movie S1). Such configuration generates anisotropic thermal diffusivity guiding more heat flow in high diffusivity regions (unintercalated area, Fig. 5D). We observed an increase in the temperature on the high diffusivity side shown in Fig. 5E. The temperature change can be enhanced by designing the geometry of the back electrodes (figs. S21 and S22 and movie S2). This observation suggests that the programmable in-plane thermal diffusivity can guide the in-plane heat diffusion defined by the voltages configuration on the electrodes.

## DISCUSSION

The high in-plane thermal conductivity of the graphite films together with the large tunability provides a unique combination for active thermal control systems. Although the switching speed of these devices is relatively slow (τ ~ 0.2 s), they can operate as a reconfigurable and adaptive thermal material. One immediate application would be spacecraft thermal management that relies on passive radiators to dump the excess heat generated by the electronics on the spacecraft. The switching speed of ~0.2 s in our graphite-based electrothermal switch is primarily limited by the diffusion of ions in the electrolyte and their intercalation into the graphite layers (see fig. S24). Key factors affecting this include ionic mobility, electrolyte viscosity, intercalation kinetics, and device geometry. While this response time is slower than that of some solid-state switching mechanisms, it remains suitable for spacecraft thermal management, where thermal changes occur over longer timescales, i.e., minutes, and high-speed switching is typically not required. Potential improvements include using faster electrolytes, thinner films, or engineering the graphite structure to improve ion accessibility. These avenues could make the technology more viable for dynamic thermal control scenarios in both space and electronics.

By merging the tunability of the IR emissivity with the in-plane thermal conduction can provide large modulation of heat rejection/radiation rate. Developing lighter and reconfigurable radiator would improve the energy budget of the spacecraft eliminate mechanically tunable components. The reported graphene-based thermal switch has strong environmental tolerance, making it well suited for space applications. The switching mechanism relies on the intercalation of an ionic liquid into graphene layers, a process that benefits from the inherent stability and unique thermal properties of both materials. Specifically, the ionic liquid used in our device has zero vapor pressure, eliminating concerns related to evaporation or outgassing in the vacuum of space. Additionally, the operating temperature range of the ionic liquid is compatible with the thermal environment typically encountered in satellite systems, which spans from a few degrees below zero up to ~90°C. This wide temperature tolerance enables switching operation under varying thermal loads, including day-night cycles and orbital transitions. The lack of moving parts further contributes to its robustness against mechanical vibrations and shocks, enhancing its suitability for launch and long-term operation in space environments.

Another motivating example is the reconfigurable thermal circuits, which can guide and manipulate heat flow within the plane. Area-selective intercalation can provide reconfigurable high/low–thermal conductivity regions (see fig. S23). Alternating regions of high/low thermal conductivity could also provide larger on/off ratio due to the interfacial scattering between the domains. This reconfigurable in-plane heat transport can be used as a thermal diode for heat scavenging (*1*). The thickness of the active graphene layer limits the total conductance. By stacking and folding method, the thermal conductance of the films can be enhanced by orders of magnitude. One such configuration would be a scrolled cylinder that could function as a tunable heat pipe.

In summary, our results demonstrate that the electrical controllability of thermal conductivity of graphite films enables a practical realization of electrothermal switches, outperforming the existing literature (benchmarking studies, section S1). We anticipate that these proof-of-concept devices may pave a realistic pathway for a new class of active thermal devices, which can be used for energy harvesting, thermal management or thermal circuits. Additional to the tunable thermal conductivity, these devices yield IR emissivity modulation (*28*, *29*). This dual functionality would enable more flexibility for designing adaptive thermal management systems for space applications.

## MATERIALS AND METHODS

### MLG synthesis

The MLG (polycrystalline graphite thin film) was synthesized by CVD (planarTECH LLC) on 25-μm-thick nickel foils (Alfa Aesar, 12722) following the procedure reported in (*29*). The nickel substrate was etched in 1 M FeCl_3_ solution in ~8 hours, and the MLG on membranes were rinsed with deionized water.

### Devices fabrication

The devices have three-layer structures as the MLG graphene as anode, a porous (43%±5) polyethylene (PE) 5-μm-thick membrane (GELON lib) acted as a separator soaked with ionic liquid (DEME-TFSI, Sigma Aldrich), and the back electrode as a 60-nm platinum sputtered on the other side of the PE substrate.

### SThM devices

SThM devices follow a similar structure used in previous devices, with the only difference of the top electrode made by highly oriented pyrolytic graphite. Those enable to have a smooth surface that is compatible with thermal AFM analysis. At least three samples were characterized and tested.

### Thermal imaging setup

The setup uses a pulsed (1 Hz) tunable laser (SuperK COMPACT NKT) and HD IR camera (FLIR T440) mounted with a set of macro lenses (50 or 25 μm). The videos were recording at a speed of 30 frames per second.

### Laser-based MTR

The pump and probe beams were derived from two OBIS Coherent continuous wave lasers with a wavelength of 637 and 785 nm, respectively. The pump laser output was modulated using KEYSIGHT Waveform Generator varying between a frequency range of 1 to 5 kHz. To spatially scan the pump beam on top of the sample, Newport ESP301 Motion Controller was used in conjunction with the stage containing the sample. Both the pump and probe beams were focused on the sample with a spot size of ~1 μm using a 50× microscope objective. Last, the Stanford Research Systems Lock-in Amplifier was used to calculate the phase lag as a function of the distance between pump and probe. The model thermal wave profiles were calculated and fitted with the experimental data using MATLAB.

### Scanning thermal microscopy

The MLG and graphite samples were grounded to avoid any parasite current, which could interfere with the measurements. Before each SThM measurement, the tip resistance was matched with the variable resistor in the Wheatstone bridge to maximize its accuracy. The AFM maps were processed using Gwyddion software using standard fitting process and denoising operations. The SThM data were offset by shifting the minimum value to zero, enabling a qualitative comparison.

### X-ray photoelectron spectroscopy

The XPS analysis was performed using a Thermo Fisher K-Alpha spectrometer.

### Scanning electrons microscopy

The MLG sheet grown onto nickel foil was imaged under scanning electron microscopy (SEM) using a Hitachi SU5000 SEM.

### X-ray diffraction

The XRD characterization was carried out in situ under ambient conditions using a Rigaku SmartLab x-ray diffractometer. Data were collected from θ/2θ scans using Cu-Kα radiation (λ = 1.540526 Å). For each scan, a bias voltage (0.1-V step) was applied to the device with a Keithley 2400 source meter.

### UV-visible

Ultraviolet (UV)–visible measurements were carried out using a Thermo Scientific Evolution 201 spectrometer using a xenon flash lamp in a double beam geometry. A bandwidth of 1 nm with a 0.12-s integration time was used. MLG membranes were cast across a 10-mm-diameter hole in a rigid plastic sheet to create a freestanding membrane through which the beam was passed to determine optical transmittance with removed effect of substrate.

### Conductivity measurements

The sheet resistance measurements were carried out using Keithley 2400 source meters and a Keithley 2110 digital multimeter. The source meter was used to intercalate the device with a voltage step of 0.1 V, where the multimeter was used for the four-point probe configuration.

### Raman spectroscopy

Raman measurements were carried out using a Renishaw Raman spectrometer using a 633-nm laser, 50× objective, at 50% laser power, and 3-s accumulation time.

### Differential scanning calorimetry

Differential scanning calorimetry (DSC) measurements were carried out on the NETSZCH STA Jupiter 440 F5. The instrument was calibrated across its working range (20° to 1600°C) using indium, zinc, aluminum, gold, and palladium metals. An 85-μl corundum (Al_2_O_3_)–lidded crucible was used for the calibration and subsequent measurements. The DSC measurements to find the specific heat capacity ( $C_{p}$ ) were carried out using the IsoStep method, and the same crucible was used for the baseline (i.e., blank), reference (synthetic sapphire crystal), and sample measurements.

## Supplementary Materials

### The PDF file includes:

Sections S1 to S12 Figs. S1 to S25 Legends for movies S1 and S2 Tables S1 to S8 References
