# Supplementary material for: Electrically controlled heat transport in graphite films via reversible ionic liquid intercalation
Source: Sci Adv. 2025 Jul 25;11(30):eadw8588. doi: 10.1126/sciadv.adw8588 (PMC12292840; doi:10.1126/sciadv.adw8588)
Supplement: Supplementary file 1 — Sections S1 to S12 Figs. S1 to S25 Legends for movies S1 and S2 Tables S1 to S8 References [file sciadv.adw8588_sm.pdf]

Science Advances
AAAS

Supplementary Materials for

Electrically controlled heat transport in graphite films via reversible ionic liquid intercalation

Pietro Steiner *et al.*

Corresponding author: Coskun Kocabas, coskun.kocabas@manchester.ac.uk

Sci. Adv. **11**, eadw8588 (2025)
DOI: 10.1126/sciadv.adw8588

The PDF file includes:

Sections S1 to S12
Figs. S1 to S25
Legends for movies S1 and S2
Tables S1 to S8
References

Other Supplementary Material for this manuscript includes the following:

Movies S1 and S2

1. Materials and Methods

Determination of MLG film Thickness: The multilayer graphene thickness is estimated by measuring the UV-Vis transmittance of a free-standing MLG and modelling the optical properties of the as-deposited (unintercalated) films. The optical modelling accounts for the intraband (Drude) and interband contributions to the optical conductivity. The following parameters used for the modelling are the lattice dielectric constant of 5, the Fermi level of 0.1 eV, and lattice scattering constant of 1.5 fs(*34*). These parameters agree with the previously reported values for undoped graphene(*42*). Following evaluation of the optical conductivity, dielectric constant, and complex refractive index of undoped graphene, the reflectance, transmittance and absorbance are calculated as functions of film thickness ranging from 0 to 500 layers using the transfer matrix method (Fig. S1a). The calculations accurately estimate the absorbance of suspended single-layer graphene as 2.3 %. Finally, the measured transmittance of the MLG film was overlayed on the resulting calculated transmittance vs MLG thickness curve to extract the film thickness (Fig. S1b). The MLG thickness was estimated to be 297 layers which correspond to ~100 nm using the interlayer distance measured via the XRD (0.336 nm).

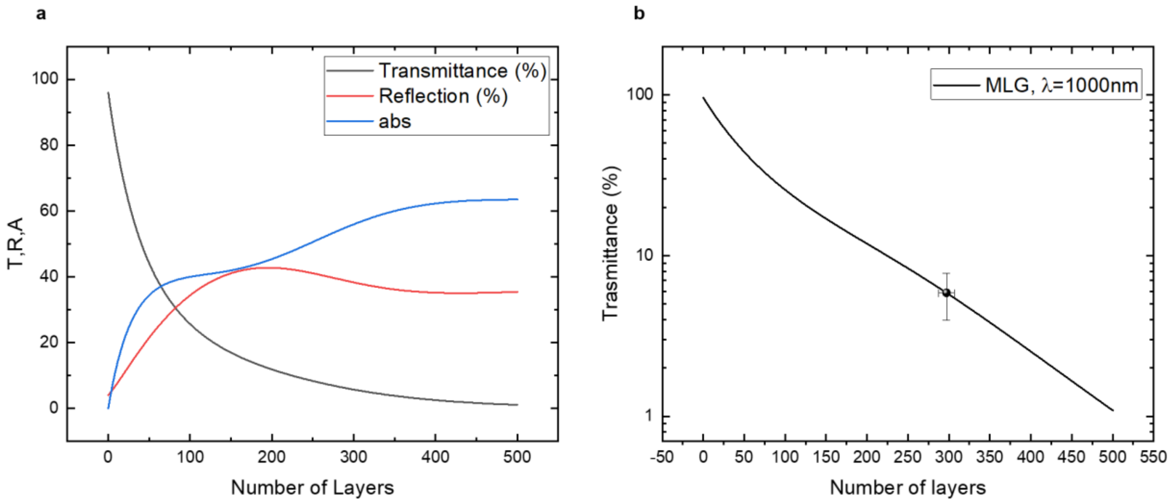

Figure S1. **Determination of MLG film Thickness. a,** Calculated transmittance of graphene film at the wavelength of 1000 nm as a function of the film thickness. A semi-infinite substrate with a refractive index of 1.5 is used in the calculations. **b,** The dot is the measurement result. The error bars represent the variation in the transmittance within the 100 nm range around the wavelength of 1000 nm.

Characterization of grain size: The grains size distribution was calculated via scanning electron microscopy (SEM images (Fig S2 a-c). the grain lateral size distribution is reported in Figure S2 d which displays an average grains size of 1.2 $\pm$ 0.5 μm.

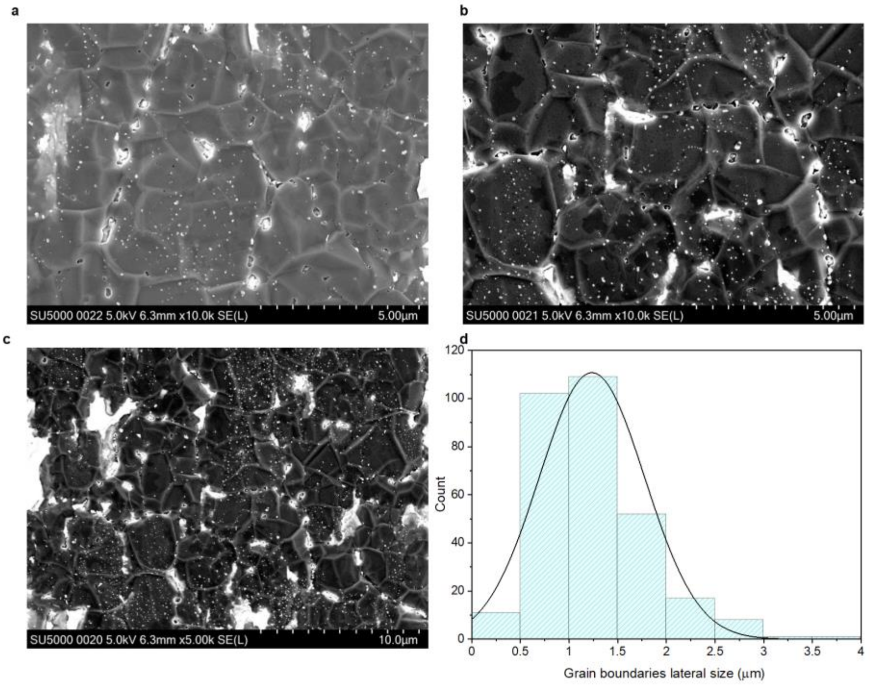

Figure S2. **SEM of graphite film.** SEM images (**a-c**) of the Multi-layer graphene grown onto Nickel foil. **d,** histogram distribution of the later size grains.

Supplementary Video 1 Heat Steering Device:

The Thermogram video displays the real-time heat steering capabilities of the device. Ten back electrodes were used to intercalate the device locally, producing programmable in-plane thermal diffusivity anisotropy, which enables to guide the heat in the desired direction preferentially. A pulsed laser (1 Hz) is used as a heat source.

Supplementary Video 2 Heat Channel Device:

The Thermogram video displays the heat guiding capabilities of the channel device. The heat provided by a continuous laser is channelled by intercalating the whole device, excluding a small portion of it.

2. **Benchmarking Studies**

We performed benchmarking studies of our thermal modulation scheme against existing tuneable materials. The key metrics for thermal applications are initial thermal conductivity, modulation, modulation speed and external stimulus. As outlined below, the devices presented in this manuscript have superior performance for these parameters compared to all the induced electrical stimulus thermal conductivity modulation materials. Additionally, our technology devices outperformed the current state of the art in terms of higher initial thermal conductivity, which is a key parameter to any real-world application.

Electrical Stimulus									
Name	Thermal conductivity (W m^{-1} K^{-1})	Modulation	External stimulus	Speed	Operative Temperature	Additional information			Reference
Multi layers Graphene	1104-80	13.8 fold	0-3V	~0.2s	RT	⬇	=	↻	This work
$SrCoO_x$	BM-SCO =1.7<br>P-SCO = 4.3<br>H-SCP= 0.44	2 fold<br>9.8 fold	0<br>±4V	~1 min<br>~30 mins	RT	⬆	/	↻	43
TiS_2	4.45 to 0.69	6.5 fold	1.8V *chemical reaction	/	/	⬇	=	/	44
InAs nanowires	0.2-0.5	2.5 fold	0<br>±10V	/	50K	⬆	/	/	45
MOS_2	105 to 45	2.3 fold	0-3V	/	RT	⬇	=	/	12
Suspended Graphene	~2 to 4.3 *Electrical conductity	2 fold	0<br>±5V	/	100K	⬆	=	/	9
MoS_2	45 to 85	1.9 fold	0-3V	14 mins	RT	⬇	⊥	↻	20
$LiCoO_2$	5.4 to 3.7	1.5 fold	0-4.5V	~6 h	RT	⬇	=	↻	21
Black Phosphorous	86 to 63<br>*zz direction	1.4 fold	0-2V	~3 h	RT	⬇	=	↻	19
Graphene	/	1.3 fold	0-4V	~30s	RT	⬆	⊥	↻	14
PZT	1.44 to 1.62	1.1 fold	100kV/cm	~10s	RT	⬆	/	↻	46
$Pb(Zr_{0.3}Ti_{0.7})O_3$	~1.15 to 1.02	1.1 fold	0-10 V	~1-10s	RT	⬇	/	↻	47
Graphene encapsulated h-BN	8 nW/mk to 1.5 nW/mk Ke	5.3 fold	0- 0.5 V<br>*charge neutrality	/	75K	⬇	/	/	48
P(VDF-TrFE)	0.2 to 0.3	1.5 fold	Electric field (80 MV/m)	/	300K	⬆	/	/	49

Table S1: Benchmark table of electrical stimulus thermal conductivity modulation materials

Legend:	
=	In-plane thermal conductivity
⊥	Out of plane thermal conductivity
⤷	Decreases the thermal conductivity
⤴	Increases the thermal conductivity
↻	Reversible process

Magnetic Stimulus									
Name	Thermal conductivity (W m^{-1} K^{-1})	Modulation	External stimulus	Speed	Operative Temperature	Additional information			Reference
$Co_3V_2O_8$	~1 to ~100	100 fold	0-14T	/	7K	⬆	/	/	50
$HoMnO_3$	~1 to ~20	20 fold	0-14T	/	4.1K	⬆	/	/	51
Ni nanowires	~32 to ~12	2.7 fold	0-0.1T	/	RT	⬇	/	/	52
InAs nanowires	0.5 to 0.2	2.5 fold	0-0.6T	/	50K	⬇	/	/	45
Co/Cu	~18 to ~32	1.8 fold	0-0.2T	/	RT	⬆	⊥	/	53
LCNs	0.34 to 0.22	1.5 fold	0-0.4T	25min	423K	⬇	/	↻	54

Table S2: Benchmark table of magnetic stimulus thermal conductivity modulation materials

Temperature Stimulus:

Name	Thermal conductivity ($W\ m^{-1}\ K^{-1}$)	Modulation	External stimulus (K)	Speed	Operative Temperature (K)	Additional information			Reference
VO_2 nanobeams	~1 to ~7	70 fold	±10	/	340	⬆	/	/	55
PE Nanofiber	~ 20 to 2	10 fold	±20	/	440	⬇	/	↻	56
$Al_{61.5}Cu_{26.5}Fe_{12}$	~1 to ~9	9 fold	±350	/	650K	⬆	⊥	/	57
$Ge_2Sb_2Te_5$	Three phases: 0.25 to 0.45 to 1.32	5.2 fold	±135	/	435	⬆	/	/	58
H_2O	~2.5 to 0.5	5 fold	±10	/	273	⬇	/	↻	59
V/VO_2	1.51 to 4.87	3.2 fold	±10	/	340	⬆	/	/	60
Graphite/hexadecane	~1.2 to 0.4	3.2 fold	±1	/	291.15	⬇	/	↻	61
CNT/Hexadecane Composites	0.51 to 0.17	3 fold	±1	/	291.15	⬇	/	↻	62
Ag_2Te	1.3 to 0.5	2.6 fold	±20	/	423 K	⬇	⊥	/	57
VO_2	~3 to 6	1.8 fold	±10	/	343.15	⬆	/	↻	63
Ni-Mn-In Heusler alloy	7/8.5 to 11.5/13	1.75 fold	±25	/	325	⬆	/	/	64
Gadolinium	~14.5 to ~10	1.4 fold	±175	/	292 K	⬇	/	/	65
$Mn_{1.014}NiGe$	11 to 15.5	1.4 fold	±25	/	600	⬆	/	/	66
$Mn_{1.007}CoGe$	7 to 8.5	1.2 fold	±25	/	520	⬆	/	/	66
PNIPAM	~0.61 to 0.53	1.15 fold	±6	/	303 K	⬇	/	/	67

Table S3: Benchmark table of temperature stimulus thermal conductivity modulation materials

Additional Thermal Properties Modulation:<br>Thermal diffusivity, thermal conductance and thermal resistance									
Name	Thermal properties	Modulation	External stimulus	Speed	Operative Temperature	Additional information			Reference
Graphene	2000 to 70000 (cm^2/s)<br>*nanoscale	35 fold	Electrical stimulus	Fs window	RT	⬆	=	/	24
Graphene Foam	~ 50 to ~ 360 (W/m^2K)	~8 fold	Pressure<br>(0-140 Kpa)	10 mins	RT	⬆	⊥	↻	68
VO_2 Nanobeam	~22 to ~34 (nW/K)	1.6 fold	Temperature<br>(±60 K)<br>0.047 V	/	340 K	⬆	/	↻	69

Table S4: Benchmark table of additional stimulus thermal conductivity modulation materials

Other Stimulus: Pressure; Hydration; Light									
Name	Thermal conductivity (W m^{-1} K^{-1})	Modulation	External stimulus	Speed	Operative Temperature	Additional information			Reference
Squid ring teeth-based bio-polymers	0.3 to 1.3	4 fold	Hydration	1min	RT	⬇	/	↻	15
$LiBH_4$	0.9 to 3.3	3.6 fold	0.1-0.9GPa	/	~300K	⬍	/	/	70
Azobenzene	0.35 to 0.1	3.5 fold	Radiation: 375/530 nm 630 mWcm^{-2}	10 s	RT	⬍	⊥	↻	71
NaCl	1.6 to 2.9	1.8 fold	19-29 GPa	/	/	⬍	/	/	72
Polycrystalline graphene	551 to 395	1.4 fold	1% uniaxial strain	/	RT	⬍	═	/	73

Theoretical studies									
Name	Thermal conductivity (W m^{-1} K^{-1})	Modulation	External stimulus (K)	Speed	Operative Temperature (K)	Additional information			Reference
Polyethylene Nanofiber	~ 50 to ~4.3	12 fold	Temperature and/or Strain	/	/	/	/	↻	56
Graphene/MoS_2 Heterostructure	~9.5 to 1	9.5 fold	Strain (15%)	/	/	/	/	/	74
Barium Titanate	~22 to ~2.3	9.4 fold	Electric field + induced polarization	/	450K	/	/	/	75
Graphene	/	8 fold	Pressure (50 Gpa)	/	300K	/	/	/	76
Graphite	1232 to 444	2.8 fold	intercalation	/	/	/	═	/	40
Bilayer Graphene	100 to 55	1.8 fold	Strain (15%)	/	/	/	/	/	74
Bilayer MoS_2	35 to 20	1.7 fold	Strain (15%)	/	/	/	/	/	74
perovskite $PbTiO_3$	~16 to ~13	1.2 fold	Ferroelectric domain walls	/	200K	/	/	/	77

Table S5: Benchmark table of theoretical thermal conductivity modulation materials

3. **Thermal Wave Microscopy**

Thermal wave microscopy (TWM) utilised a periodic heat source to generate thermal waves, which diffuses within the material. The thermal wave displays a specific temporal and spatial profile related to the specimen thermal conductivity and volumetric heat capacity. The penetration depth of the thermal wave is defined as the propagation distance of the thermal wave within a temporal period. It is intrinsically related to the heat source's modulation frequency and defines the spatial resolution of the thermal measurements. Thermal wave microscopy can be considered a thermal transient characterisation technique; therefore, the thermal gradient is recorded as a function of the time, providing information on the materials' heat propagation velocity (thermal diffusivity). The conversion of thermal diffusivity into thermal conductivity requires the independent measurement of specific heat capacity (Cp) and mass density (ρ_m).

In this work, two TWM setups have been exploited to characterise the device: a laser-based modulated thermal reflectance microscopy (MTR) and infrared thermography. MTR characterisation enables to probe only the top layer of the device due to the kHz heat source modulation, high spatial resolution (µm) and low penetration depth. Furthermore, the infrared thermography setup was used to characterise the thermal conductivity of the whole device due to its high penetration depth thermal waves, which propagated in the mm scale (main text Figure 2d).

3.1 Infrared Thermography

The thermal imaging setup follows the angstrom method, where a periodic heat oscillation is used to produce a thermal wave that diffuses into the sample(*78*) In our setup, the heat source is a tuneable pulse laser. The detector is a high-resolution infrared camera that provides accurate temperature measurements with a pixel size resolution of up to 25 µm (Fig. S3).

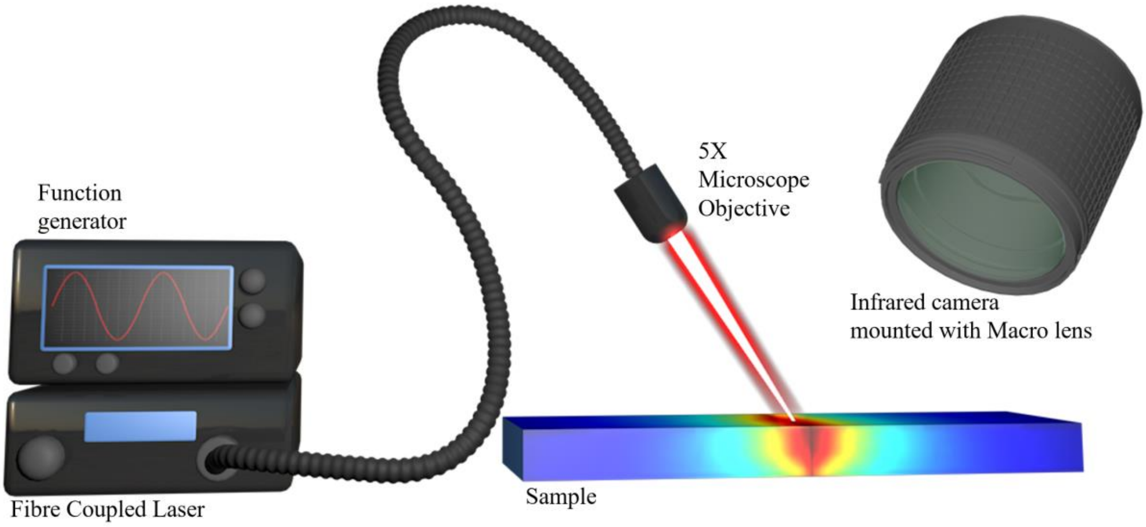

Figure S3. Schematic representing the infrared thermography setup. The thermal source is a broadband laser beam modulated at one hertz, which is focused on the sample surface. The diffusive thermal waves are recorded via a high-resolution infrared camera.

Transient thermal phenomena are well described by the diffusion equation(*79*) (1).

$$\frac{\partial \delta T(t,\bar{r})}{\partial t} - D\nabla^2 \delta T(t,\bar{r}) = \frac{q(t,\bar{r})}{c} \quad \text{(S1)}$$

Where:

δT

$= Temperature\ variation\ above\ the\ T\_0 in\ which\ the\ experiment\ is\ performed\ (T\ ambien$

$r = \{x_1, x_2, x_3\} e = Represent\ the\ spherical\ radial\ coordinate$

$q = Absorbed\ power\ density$

$D = Thermal\ diffusivty$

$c = Volumetric\ specific\ heat\ capactiy$

The thermal diffusivity and the specific heat capacity are both functions of the temperature. However, a general condition is that δT << T, therefore, we can assume $(T + \delta T) \approx c(T)$ and $D(T + \delta T) \approx D(T)$.

Considering the mm scale heat propagation and the micron laser spot size, the laser can be approximate as a point source. Therefore, the absorbed power density can be written as(*79*):

$$q(t, \overline{r}) = P_0 e^{-i\omega t} \delta^3(\overline{r}) \tag{S2}$$

3.1.1 Semi-infinite Model

The semi-infinite isotropic model considers that the heat propagates in spherical heat diffusing waves(*79*), which can freely propagate throughout the materials. The general solution of the differential equation (1) is in the form of a periodic damping wave(*79*):

$$\delta T = A_0 e^{-kr} e^{-ikr} e^{-i\omega t} = \frac{q(t, r)}{c} \tag{S3}$$

Where:

$A_0 e^{-kr} = Amplitude\ coefficient, exponential\ decay\ function$

$e^{-ikr} = periodic\ function\ in\ r$

$e^{-i\omega t} = periodic\ function\ in\ t$

The following equation represents the response δT as a function of frequency and space ($\delta T(w, \overline{r})$).

$$\delta T(w, r) = \frac{P_0}{k} \frac{1}{r}\ exp\left(-\sqrt{\frac{\omega}{2D}} r\right) exp\left(-i\sqrt{\frac{\omega}{2D}} r\right) \tag{S4}$$

Although both amplitude and phase give information regarding the thermal diffusivity of the materials, using the phase provides several advantages, such as independent by the laser fluctuation(*79*) and heat losses(*78*).

The thermal diffusivity can be determined by keeping the modulation laser frequency constant and measuring the phase shift of the diffused thermal wave as a function of the distance(*78*) This method of calculating the thermal diffusivity is also preferable due to the nature of our thermal setup, which enables accurate spatial temperature measurement.

$$D = \frac{\omega}{2k_r^2} \tag{S5}$$

Where:

k_r = *wavenumber*

ω = *laser pulsed frequency*

The Semi-infinite model can be used to determine the effective thermal diffusivity for a finite thickness model, which can be subsequently converted into actual thermal diffusivity.

3.1.2 Finite Sample Thickness Model:

To take into account, the finite sample thickness (5µm) we approximate the device as one homogeneous layer. The diffusion equation becomes the following[50]:

$$T(r) = \int_0^\infty \frac{P_0 \cosh(\eta\, d)}{k\eta \sinh(\eta d)} \mathrm{J}_0(\xi r)\xi\, d\xi \qquad \text{(S6)}$$

$$\eta^2 = \xi^2 + \frac{i\omega\rho C}{k} \qquad \text{(S7)}$$

This has a solution of the form $A\, exp(imr)$ where m is the slope in the phase profile and both A and m are functions of conductivity (k), density (ρ), heat capacity (C), frequency (ω) and diffusivity (D).

Solving the equations enables the conversion of the effective diffusivity into actual thermal diffusivity (Fig. S4.)

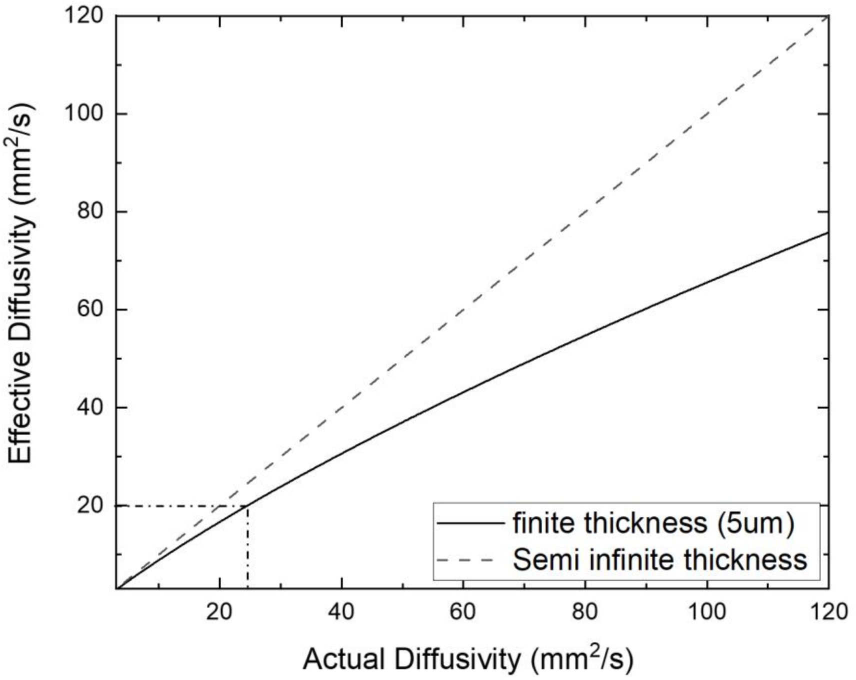

Figure S4. Thermal diffusivity conversion curve. The solid line represents the conversion curve calculated considering the finite device thickness (5 µm). The dotted line represents the semi-infinite model, which considers infinite the device thickness.

3.1.3 Data Acquisition and Processing

Each thermal diffusivity measurements consist of a 20-second video. The frames were subsequently processed using a lock-in algorithm to maximise the signal-to-noise ratio and extrapolate the intensity and the phase shift of the diffusive thermal wave (Fig. S5).

The phase shift experimental data were fit in one direction using the iterative general Least Square method and the Levenberg-Marquardt method via the LabVIEW program. The thermal diffusivity was calculated following the model described in the section above.

The weighted mean square error of the fitted model was used to calculate the error of the thermal diffusivity.

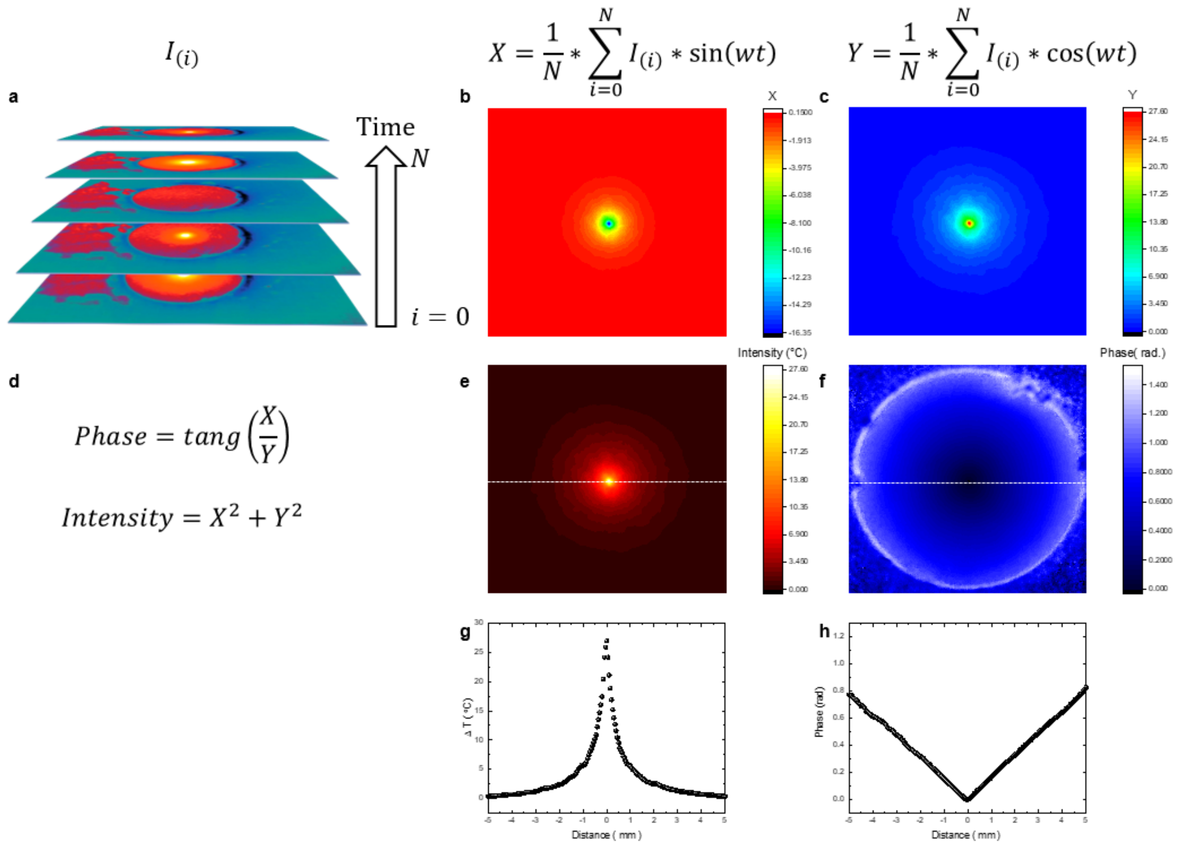

Figure S5. Lock-in image processing algorithm. **a,** A 600 frames batch were recorded and individually analysed using a lock-in based technique. **b, c,** Images X and Y calculated following the equation above (X and Y). **d,** The equations used to determine the intensity and the phase shift images**. e, f,** The intensity and the phase shift data of the periodic heatwave. **g, h,** Line profile acquired along the horizontal direction, highlighting the exponential decay nature of the intensity and the linearity of the phase shift as a function of the distance. The slope of the phase shift as a function of the distance represents the wavenumber k.

3.2 Laser-based Modulated Thermoreflectance (MTR)

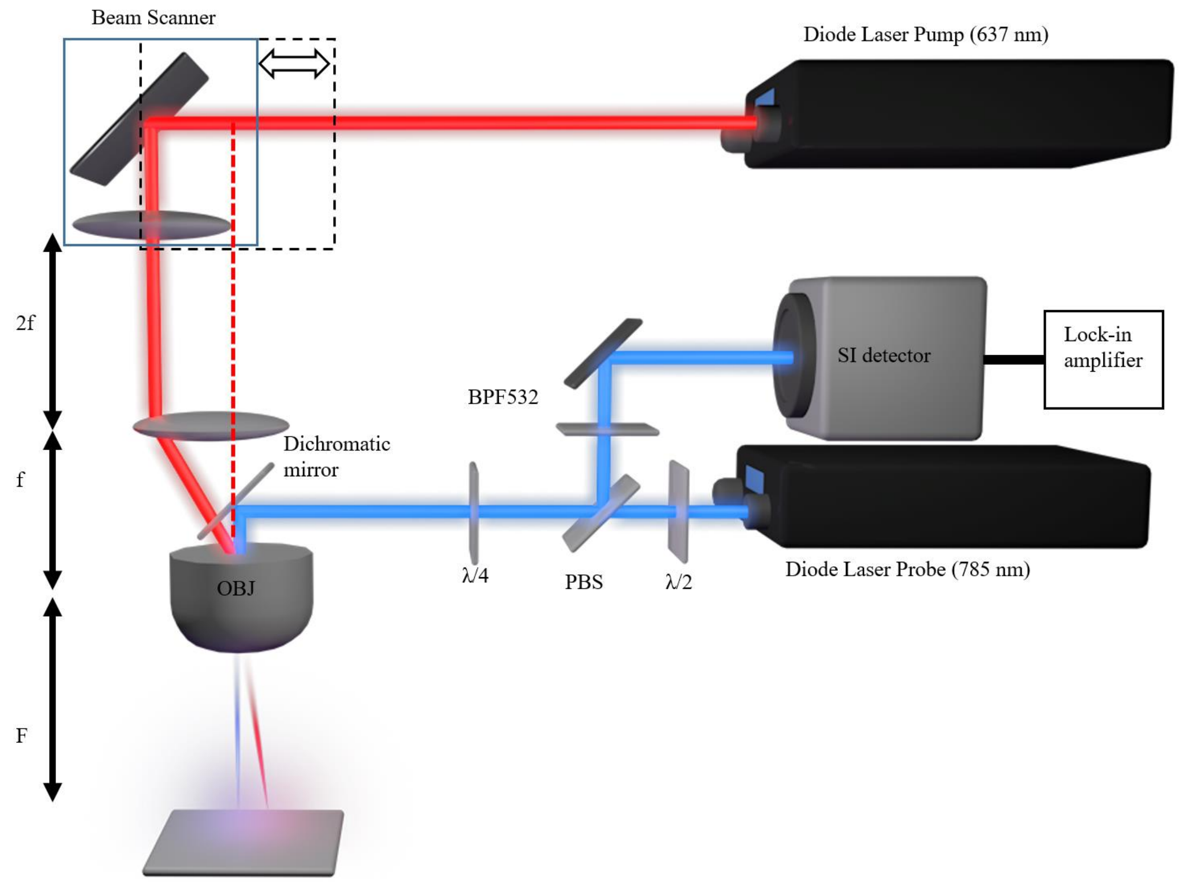

Figure S6. Schematic of the laser-based modulated thermoreflectance (MTR) system. Two continuous-wave diode lasers are used as pump and probe. The pump laser is amplitude modulated in the range of 1 KHz -5 KHz and focused onto the sample using a 50x microscope objective after being sent through pair of confocal lenses. The entrance angle of the pump beam into the objective is controlled by this lens pair. This change in entrance angle is converted to the relative position change of the pump beam with respect to the fixed probe beam at the focal plane. The probe beam is passed through the optical axis of the objective and onto the sample. The reflected beam is guided through a long wavelength pass filter that block the pump beam and into a photodiode where the signal is collected. Finally, the phase shift is measured by sending the signal to a lock-in amplifier which is in phase with the pump laser.

The Laser-based modulated thermoreflectance setup follows a pump-probe configuration (Fig. S6). A 637 nm modulated pump laser is used to heat the sample locally. The 785 nm probe laser

enables monitoring of the induced temperature changes due to modulation of the specimen reflective index. This unique setup detects the amplitude and phase of the temperature profile by laterally scanning the pump beam across the sample. The phase shift is measured as a function of the distance between the pump and probe beam. To determine the thermal conductivity, the experimental data is fitted to a theoretical model based on the following anisotropic continuum heat diffusion equation for multilayer system(*32*).

$$\rho_n C_n \frac{\partial T_n}{\partial t} = k_{zn} \nabla_z{}^2 T_n + k_{rn} \nabla_r{}^2 T_n + Q e^{i2\pi f t} \tag{S8}$$

Where:

T_n = *temperature of the nth layer.*

ρ_n, C_n = *density and specific heat of the nth layer.*

Q = *Heat source*

f = *Modulation frequency*

Subscript r and z represent the spatial coordinates in the in-plane and cross-plane directions, respectively, to address the anisotropic nature of thermal conductivity in graphene. For a multilayer material stacking, as in the case of the device, the solution is defined by a matrix approach using Feldman's algorithm(*80*).

$$\begin{bmatrix} L^+ \\ L^- \end{bmatrix}_n = \frac{1}{2\varepsilon_n} \begin{bmatrix} exp(-\mu_n d_n) & 0 \\ 0 & exp(\mu_n d_n) \end{bmatrix} \begin{bmatrix} \varepsilon_n + \varepsilon_{n+1} & \varepsilon_n - \varepsilon_{n+1} \\ \varepsilon_n - \varepsilon_{n+1} & \varepsilon_n + \varepsilon_{n+1} \end{bmatrix} \begin{bmatrix} L^+ \\ L^- \end{bmatrix}_{n+1} \tag{S9}$$

Where:

n = *subscript for the number of layers*

d_n = *thickness of the nth layer.*

$\mu_n = \sqrt{\frac{k_{r,n}}{k_{z,n}} \xi^2 + \frac{i\omega\rho C}{k_{z,n}}}$

$\varepsilon_n = k_{z,n} \mu_n$

L^+ and L^- are related to the exponentially growing and decaying terms along z-axis and for the last infinte layer, N they are defined as,

$$\begin{bmatrix} L_N^+ \\ L_N^- \end{bmatrix} = \begin{bmatrix} exp(-\mu_N\, d_N) \\ exp(\mu_N\, d_N) \end{bmatrix} \tag{S10}$$

The frequency domain solution accounting for the top transparent layer is described as,

$$\tilde{T}_1(\xi) = \frac{L_1^+ + L_1^-}{L_1^- - L_1^+}\frac{1}{2\varepsilon_1}\frac{(L_1^+ + L_1^-)\,cosh(\mu_u\,d_0)}{2\,(L_1^+ + L_1^-)sinh(\mu_u d_0)k_0\eta_0 + (L_1^+ + L_1^-)\,cosh(\mu_u\,d_0)k_0\eta_1} \tag{S11}$$

The surface temperature T(r) is finally expressed by the inverse Hankel transformation,

$$T(r,f) = 2\pi\int_0^\infty \tilde{T}_1 P\exp\left(\frac{-\xi^2 R_0^2}{2}\right)J_0(\xi r)\xi\,d\xi \tag{S12}$$

Where:

P = *Power absorbed*

R_0 = *objective radius*

J_0 = *zeroth order Bessel function*

For further information regarding the theoretical model and the solution to this equation, see our previous work(*31*, *33*).

To predict the thermal phase profiles of the multilayer graphene, a four-layer approximate system was modelled consistent with the device layers. The layers consist of graphene, polyethene & ionic liquid (PE + IL), platinum electrode and a semi-infinite layer of air, respectively, from top to bottom. Each layer's thickness, density, and thermal conductivity are used as input parameters in the solution to equation 6 and are listed in Table S3. Sensitivity analyses were carried out to determine the sensitivity of the MTR measurements to the different device layers at the different Laser frequencies. The phase profiles sensitivity analysis was calculated using the following equation:

$$S_\delta = \frac{\varphi(\delta + \Delta\delta) - \varphi(\delta)}{\Delta\delta\,/\,\delta} \tag{S13}$$

Where:

S_δ = *sensitivity of the phase profile.*

$\varphi(\delta + \Delta\delta)$ = *phase profile with a small perturbation to a parameter (conductivity)*

The Sensitivity analyses highlighted that at 1KHz modulation frequency, the MTR measures are mainly sensitive to the Multilayer graphene. The frequency regime below 1KHz offers very low sensitivity to the PE layer, but the phase profiles were inconsistent. However, with the increase of frequency, at 5KHz, the measures are partially influenced by the PE layer along with MLG. The model developed accounts for both the MLG and PE contributions by considering the in-plane thermal conductivity of both layers as a fitting parameter. The cross-plane thermal conductivity of graphene was assumed constant.

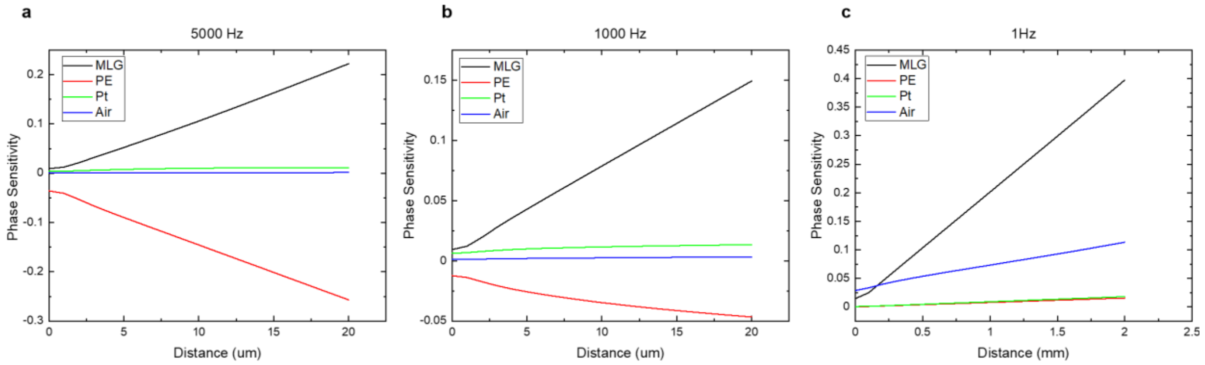

Figure S7. Sensitivity analysis. Phase profile sensitivity for all the individual layers of the device measured at a different laser modulation frequency (a: 5000 Hz; b: 1000 Hz c: 1 Hz).

A parametric study was performed by varying the thickness and the conductivity by ~10 %. Those provide helpful information regarding the actual measurements' errors. Our analysis shows that the fitted thermal conductivity values can be subject to ± 100 (W m^{-1} K^{-1}) and ± 30 (W m^{-1} K^{-1}) variation for non-intercalated and intercalated devices, respectively. The cross-plane thermal conductivity of graphene was assumed constant. To validate this assumption, a separate sensitivity analysis of the thermal wave profiles was done for cross-plane conductivity. Varying the cross-plane conductivity by ~15 % and ~80 % for the case of non-intercalated and intercalated state (predicted value of k_Z based on the thermal transport model), only resulted in 2% and 10% change in the measured in-plane conductivity using MTR. An effective diffusivity contour plot using different values of in-plane and cross-plane conductivity is shown in figure S8. This illustrates the weak sensitivity of in-plane conductivity to the cross-plane conductivity in the range of values used in this study.

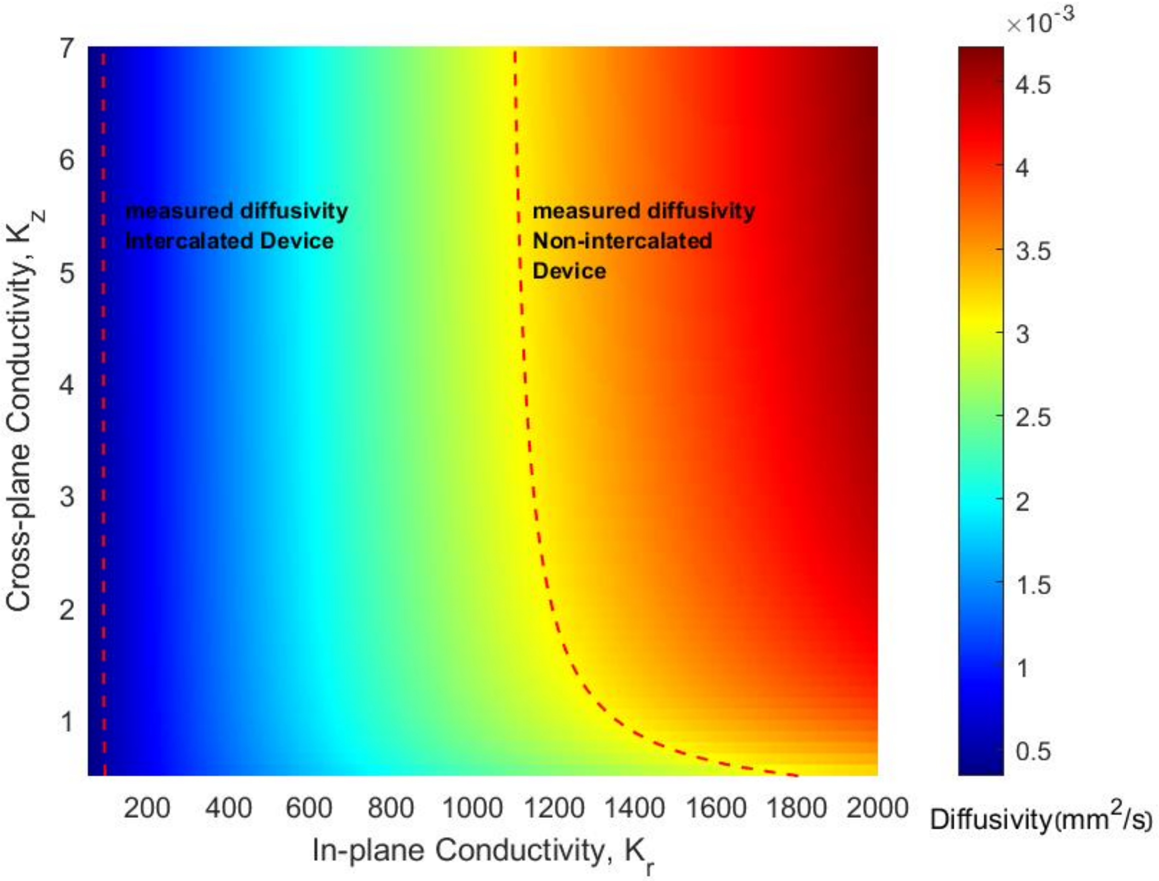

Figure S8. Cross-plane sensitivity analysis. Effective diffusivity contour plot for in-plane and cross-plane values.

A parametric study was performed by varying the thickness and the conductivity by ~10 %. Those provide helpful information regarding the actual measurements' errors. Our analysis shows that the fitted thermal conductivity values can be subject to ± 50 (W m^{-1} K^{-1}) and ± 5 (W m^{-1} K^{-1}) variation for non-intercalated and intercalated devices, respectively.

3.2.1 Cross-plane Sensitivity

The conventional TDTR method has low in-plane conductivity sensitivity and primarily measures cross-plane conductivity. In contrast, the laterally resolved methods used in this work are most sensitive to in-plane conductivity. The Sensitivity analysis was performed for the device configuration used in this work. We fitted the measured phase profiles assuming a value for cross-plane conductivity. Results are summarised below.

Cross-plane Conductivity (Assumed) W m^{-1} K^{-1}	Fitted In-plane Conductivity W/mK	
	Non-intercalated	Intercalated
6.8	1105	73
1.5	1306	80
0.5	2045	98
0.1	3750	108

Table S6: Cross-plane sensitivity analysis. The table highlights the cross-plane influence on the in-plane thermal conductivity.

These results confirm greater sensitivity to in-plane conductivity and low sensitivity to cross-plane K, particularly in the intercalated samples: Almost a two-order of magnitude change in cross-plane results in 30 % change in-plane values. Whereas, for a non-intercalated sample, an "educated assumption" is needed, as sensitivity to cross-plane conductivity is larger.

The thermal conductivity model is used for making this "educated assumption". Using this model, we perform a conservative estimate for cross-plane values for non-intercalated (6.8 $Wm^{-1}K^{-1}$) and intercalated (1.5 $Wm^{-1}K^{-1}$). Using this "educated assumption", we arrive at the highlighted values.

Table S7 displays the data utilised to convert the thermal diffusivity into conductivity.

	Thickness	Density	Specific heat capacity	Thermal Diffusivity	Thermal conductivity
units	(µm)	(g/cm^3)	(J/gK)	(mm^2/s)	($Wm^{-1}K^{-1}$)
Multi-Layer Graphene (MLG)	0.1±0.023	2.2±0.12 (*81*)	0.71 ±0.1 (*82*, *83*)		1104 ± 50 (in-plane) 6.8 (Cross-plane)
Polyethene (PE)	5±0.028	0.73±0.03	2.1±0.3 Note 1		1± 0.12 (MTR)
Ionic Liqid	-	1.4±0.1 Note 2	1.35±0.01	-	-
Polyethene + Ionic liquid (PE+IL)	5±0.028	1.416± 0.03	1.30±0.01	0.4 ±0.1	0.75±0.2
Device: (PE+IL+MLG)	5.1±0.05	1.42±0.03	1.30±0.01	15±0.3	29±2

Table S7: Physical properties of the device and individual layers necessary to calculate the thermal conductivity.

(note 1) Polyethylene - Low Density - online catalogue source - supplier of research materials in small quantities - Goodfellow. http://www.goodfellow.com/E/Polyethylene-Low-Density.html.

(note 2) Ionic liquid DEME TFSI | Diethylmethyl(2-methoxyethyl)ammonium bis(trifluoromethylsulfonyl)imide. http://www.ilschem.com/ils/showproduct.php?lang=en&id=39#ad-image-0.

(note 3)Anasys Instruments Inc, NanoTA On Afm+ and NanoIR Systems (Anasys Instruments Inc, Santa Barbara, CA 93101, Part# 00-0., 2012).

4. Reversible Intercalation:

The ionic liquid intercalation is a reversible process, by applying a positive or negative voltage, the ions can be intercalated or deintercalated. The DEME-TFSI Ionic liquid is utilized in this work, and the anion TFSI is mainly intercalated.

Figure S9a shows the thermal diffusivity as a function of the intercalation voltage. The first half cycle (black) shows the highest thermal diffusivity modulation (14 to ~7 mm^2/s). Subsequently, when the deintercalation occurs, there is an over 95% recovery of thermal diffusivity (7 to ~13 mm^2/s).

On-off cycle measurements (Fig. S9b) highlight that the device can sustain over 12 cycles without any notable thermal diffusivity modulation loss. However, stability studies (Fig. S9c) display that after 35 cycles, the off-state thermal diffusivity is substantial decreased (30%). After 65 cycles, the device loses its capability to modulate the thermal diffusivity via electrical stimulus.

The degrading of the thermal modulation proprieties can be related to the recombination of the ions of the Ionic liquid in the graphite(*28*), oxidation process and poor stability of the back electrode. Our previous works demonstrated that similar devices can be switched for over 2000 cycles(*28*, *29*). We anticipate that by optimizing the deintercalation process, improving the stability of the back electrode and protecting the active layer by oxidation process using a top polymer layer, the device cycling performance can be substantially improved. Additionally, the performance of the device can be improved by utilising different ionic liquid electrolytes. Promising results were achieved intercalating AMIM-TFSI, which almost doubled the lifetime of the device, sustaining over 100 ON-OFF cycles (Fig. S9d).

Moreover, performing the cycling test in high vacuum chamber enable to increases the device lifetime further achieving an outstanding 2000 cycles. Initial testing suggest that oxygen could be the cause of the low lifetime of the device in air (Fig. S9e). The switching speed of the device was measured by monitoring the apparent temperature of the device while the device was switched ON and OFF. We were able to achieve a fast switching speed, less 200 ms (Fig. S9e).

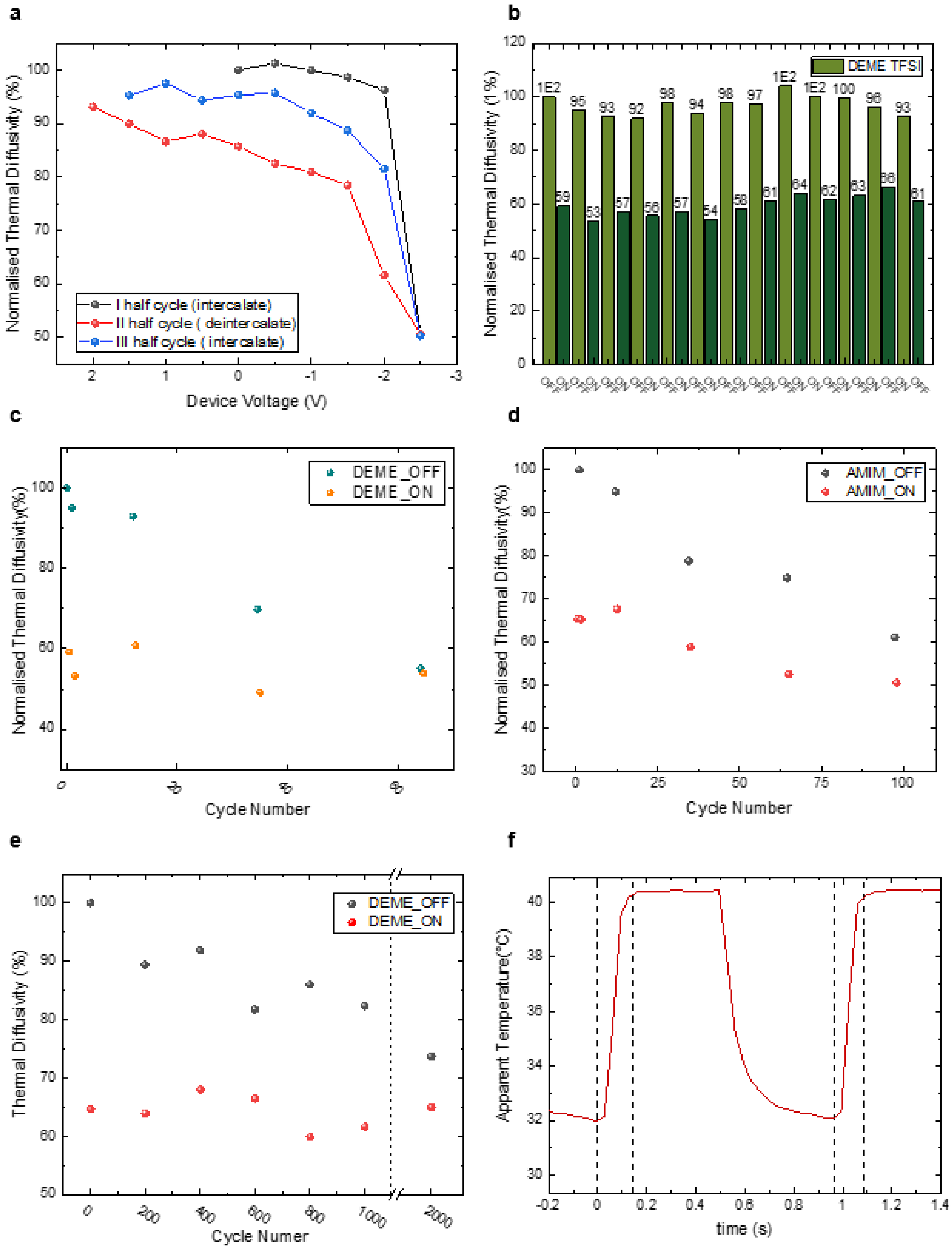

Figure S9. Reversible intercalation. a, Thermal diffusivity as a function of the intercalation process. **b**, On-off cycle measurements. **c**,**d**, Stability test for DEME-TFSI and AMIN-TFSI respectively. **e**, Stability test for DEME-TFSI performed at high vacuum 10^{-6} Torr. **f,** Speed test analysis: the switching speed is less than 200ms.

Recovering initial thermal conductivity: The reported value of ~1104 W/mK is from the MTR measurements which is after the first intercalation. This value is fully reversable. To show the reversable intercalation and recovery of the initial thermal conductivity, we repeated the experiments with MTR and IR thermograms. Figure S10shows the results before and after the intercalation. Full reversibility was achieved using both MTR and IR thermogram. The full reversibility is achievable only when the device is fully deintercalated. We observed that the initial thermal conductivity can be fully recovered after fully discharged of the device. We observed that there are fast and slow components in the discharging dynamics which is also voltage dependent. At low voltages < 2.5 V, the device can be fully recovered in seconds time scale, however V> 2.5 V, we observed a slow recovery. The device does not come back to the initial high thermal conductivity state in seconds time, however after waiting 15-20 minutes, we observed a full recovery. This slow recovery is likely due to the trapped ions inside the graphene layers.

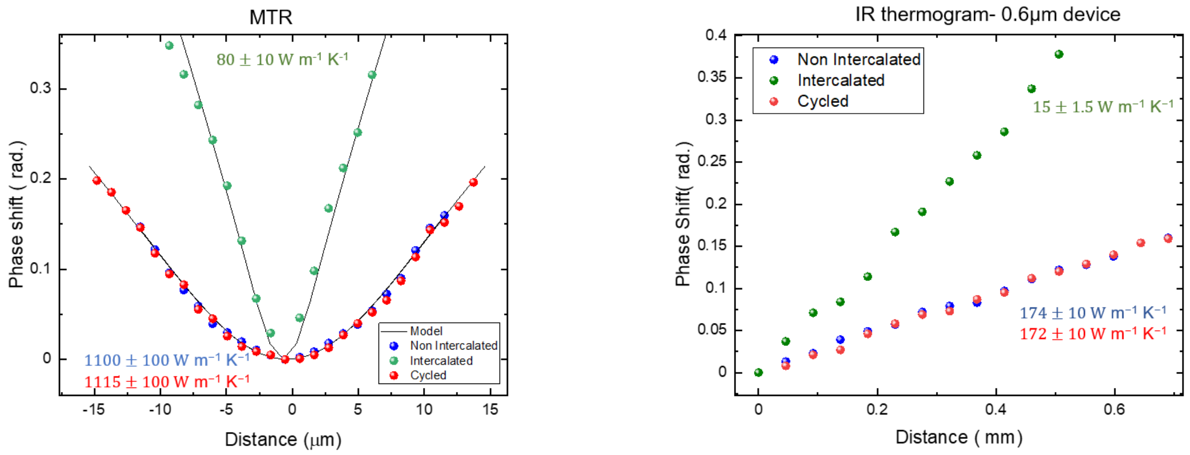

Figure S10. MTR and IR thermogram reversibility measurements. The MTR measurements displaying a 13-fold modulation, which is fully reversible after the device is fully deintercalated. Additionally, 11-fold reversible modulation were achieved using IR thermogram.

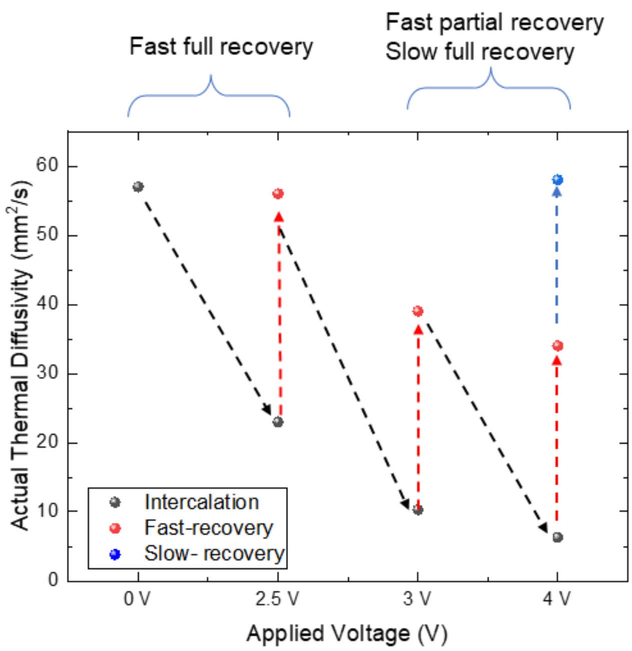

Figure S11. Recovery of thermal diffusivity. Fast and slow recovery of thermal diffusivity of the device with 1um substrate.

5. **Raman Spectroscopy**:

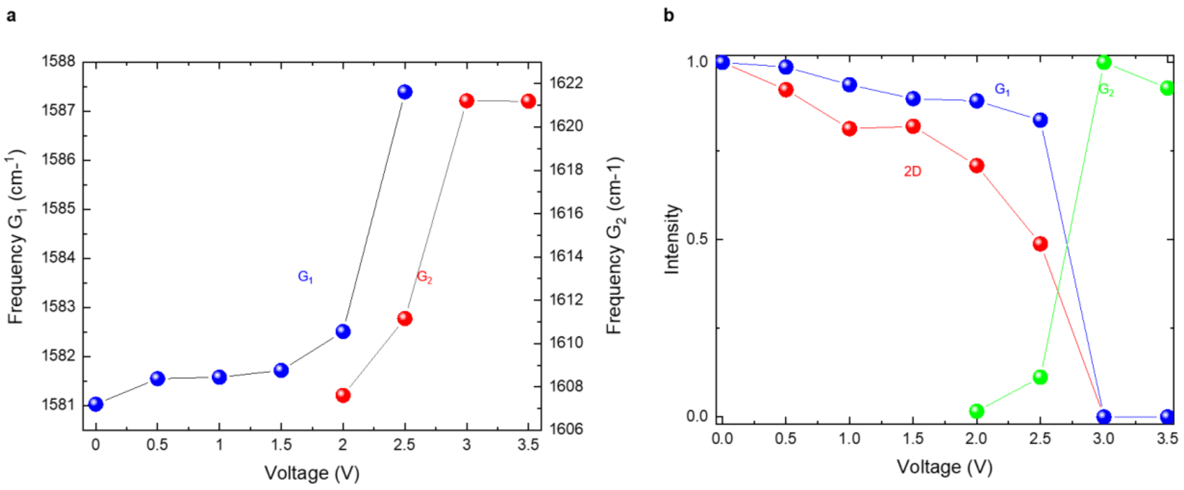

Figure S12. In-situ Raman Measurements. a, Variation of the Raman frequency of the G_1 and G_2 peaks as a function of the device voltage. **b,** Intensity of the G_1, G_2 ,2D peaks as a function of the device voltage. The intensity of 2D and G peaks diminish due to Pauli blocking condition.

6. XRD Characterisation

X-Ray diffraction allows to access the interlayer spacing in the different intercalation stages, therefore provides insights into the intercalation stage process. Intercalation of TFSI (height $d_{TFSI} =$ 8.10 nm) into MLG (interlayer spacing $c = 0.336$ nm) results in the appearance of a superlattice peak I_{00n+1} and I_{00n+2} (*35*, *36*) with varying intensity, for a Bragg angle of:

$$2\theta = 2Arcsin\frac{n\lambda}{2d_{TFSI}} \tag{S14}$$

Therefore, the ratio d_{00n+2}/d_{00n+1} will enable determination of the intercalation stage. The position and intensity of the peaks were extracted through a fit of the I(q) diffraction pattern with a Voigt function over a linear background (see Figure S13). For 00n+1, a background peak, originating from the PE substrate may influence the fit. For this reason, the background peak was fit with a Voigt function for pristine MLG and used as the background of the subsequent fits. Additionally, we note that the full width at half maximum (FWHM) of the 00(n+2) and 00(n+1) peaks presents huge variations after the intercalation process starts (see Fig. S13 c). This can be interpreted as the result of inhomogeneous intercalation that is not affecting our conclusions. We note that the FWHM of the in-plane reflections is constant throughout the process (e.g. 0.002 Å^{-1} for the 100 reflection), consistent with the in-plane vibrations remaining non-affected by the intercalation process.

For 3.4 and 3.5 V, we assess the coexistence of two distinct intercalation stages (n=2 and n=3) via the presence of two overlapping peaks for both 00(n+1) and 00(n+2). We note that this could also be the case for V_i = 2.4, 2.6 and 2.8 V, owing to the large FWHM of 00(n+2). However, this trend is not verified for the 00(n+1) reflection, possibly because of the strong PE background. Additionally, coexistence of more than two intercalation stages would result in enhanced broadening of the diffraction peaks.

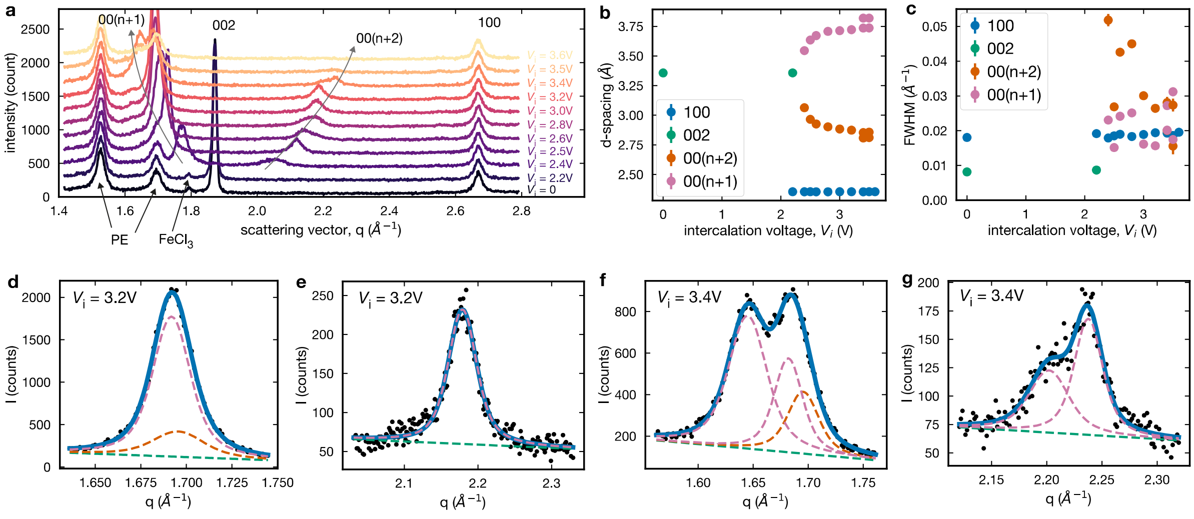

Figure S13. Fits from XRD data. a, raw XRD peaks showing PE background and 100 reflections, along with 002, 00(n+1) and 00(n+2) Bragg peaks for all intercalation voltages. **b,** d-spacing extracted from the peak position. **c,** FWHM of the diffraction peaks. **d-g,** fits of the 00(n+1) and 00(n+2) reflections with 1 or 2 Voigt functions for two characteristic spectra. The 00(n+1) peak is fit over a fixed linear (dashed green) + Voigt (dashed brown) background to account for the presence of PE. The 00(n+2) peak is fit over a linear background. The Voigt functions used in the extraction are shown as pink dashed curves, the resulting fit as a solid blue line.

Dominant stage, n	$d_{00(n+2)}/d_{00(n+1)}$ peak position ratio
1	1.5
2	1.34
3	1.25
4	1.2
5	1.17
6	1.14

Table S8: the table displays the $d_{00(n+2)}/d_{00(n+1)}$ peak position ratio of n stage graphite intercalation compound (*35*, *36*).

7. *X-ray Photoelectron Spectrometry (XPS)*

The chemical nature of the intercalant (DEME-TFSI) provides valuable insight into the intercalation process. XPS analysis displays four peaks attributed to the F1s, N^{+}1s, and N^{-}1s (Fig. S14a) .

DEME-TFSI contains two nitrogen atoms: quaternized nitrogen (DEME), which is positively charged and imide nitrogen (TFSI) which is negatively charged. The different nature of the nitrogen atoms yields two distinctive N1s peaks (Fig. S14b).

Qualitatively analysis revealed a 20 % charge imbalance at -3 V (ratio N^{-} to N^{+}) with a charge excess of approximately one ion for ~200 C atoms(*28*). As a result of the intercalation, the graphite is p-doped.

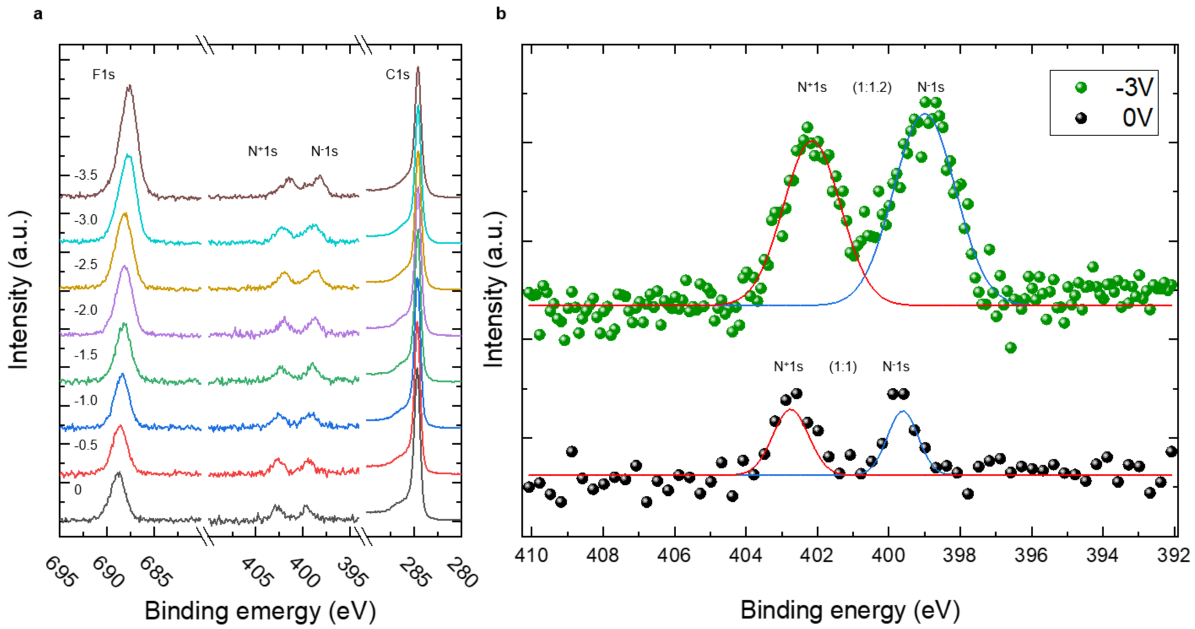

Figure S14. In-situ XPS Measurements. a, XPS measurements acquired at different device voltages, provide useful information on how the chemical composition of the device varies during the intercalation. **b,** Quantitatively analysis of N^{+}1s and N^{-}1s peaks enables to determinate the amount of charge imbalance present in the intercalated device, ~20 %.

8. **Thermal Conductivity Model:**

An analytical model within the framework of phonon-mediated thermal transport is presented. The measured values of thermal conductivity using the MTR method in conjunction with the voltage-dependent XRD and sheet resistance data are used to model in-plane and cross-plane conductivity of the MLG based on Klemens approach (*37*). We present an analytical expression for thermal conductivity by approximating the hexagonal Brillouin zone with a cylinder in the q-space. The anisotropic nature of thermal transport in graphite is accounted by including the contribution of phonons propagating along different direction. The total thermal conductivity is calculated by a summation over one longitudinal acoustic and two transverse acoustic phonon branches with linear dispersion profile, where conductivity for a single acoustic branch is defined as(*32*)

$$\boldsymbol{k}_{i(r\ or\ z)} = \frac{k_B}{8\pi^3}\int_0^{q_{i,max}}\int_0^{2\pi}\int_{-q_{z,max}}^{q_{z,max}} \frac{x^2 e^x}{(e^x-1)^2}|\vec{v}\cdot\hat{n}|^2 \tau\, q\, dq\, d\varphi\, dq_z \qquad \text{(S15)}$$

Where

i = Subscript for in-plane(r) and cross plane(z) conductivity.

q = phonon wavevector (q_z - cross-plane direction)

$x = \frac{hv_i q}{k_B T}$

k_B *= Boltzmann constant*

ħ *= Reduced Planck's constant*

τ *= Total phonon relaxation time*

$\vec{v}$ *= Phonon velocity*

$\hat{n}$ *= direction along which thermal conductivity is calculated (* $\hat{n}_{\parallel}$ in-plane and $\hat{n}_{\perp}$ cross-plane)

The integral is expressed in terms of phonon wavevector (q) spanning from the center of the Brillouin zone (G) to the edges (M, A) and azimuthal angle (φ) as shown in Figure S15. To determine the phonon velocity projection along the heat flux direction, a polar angle (θ) was also defined with respect to the z axis. The in-plane and cross-plane velocity projections are defined as,

$$v_{\parallel} = \vec{v}\cdot\hat{n}_{\parallel} = v \sin\theta \cos\varphi \qquad \text{(S16)}$$

$$v_{\perp} = \vec{v}\cdot\hat{n}_{\perp} = v \cos\theta \qquad \text{(S17)}$$

respectively, where v is effective phonon velocity in the q-space based on the Christoffel equation and is expressed as,

$$v = \left[v_{in-plane}^2 \sin^2\theta + v_{cross-plane}^2 \cos^2\theta\right]^{1/2} \quad \text{(S18)}$$

Here, the $v_{in-plane}$ & $v_{cross-plane}$ are the phonon branch dependent in-plane and cross-plane velocities. The phonon velocities as well as the linear dispersion profile used in this model are obtained from the valence force field calculations for graphene (*33*, *38*).

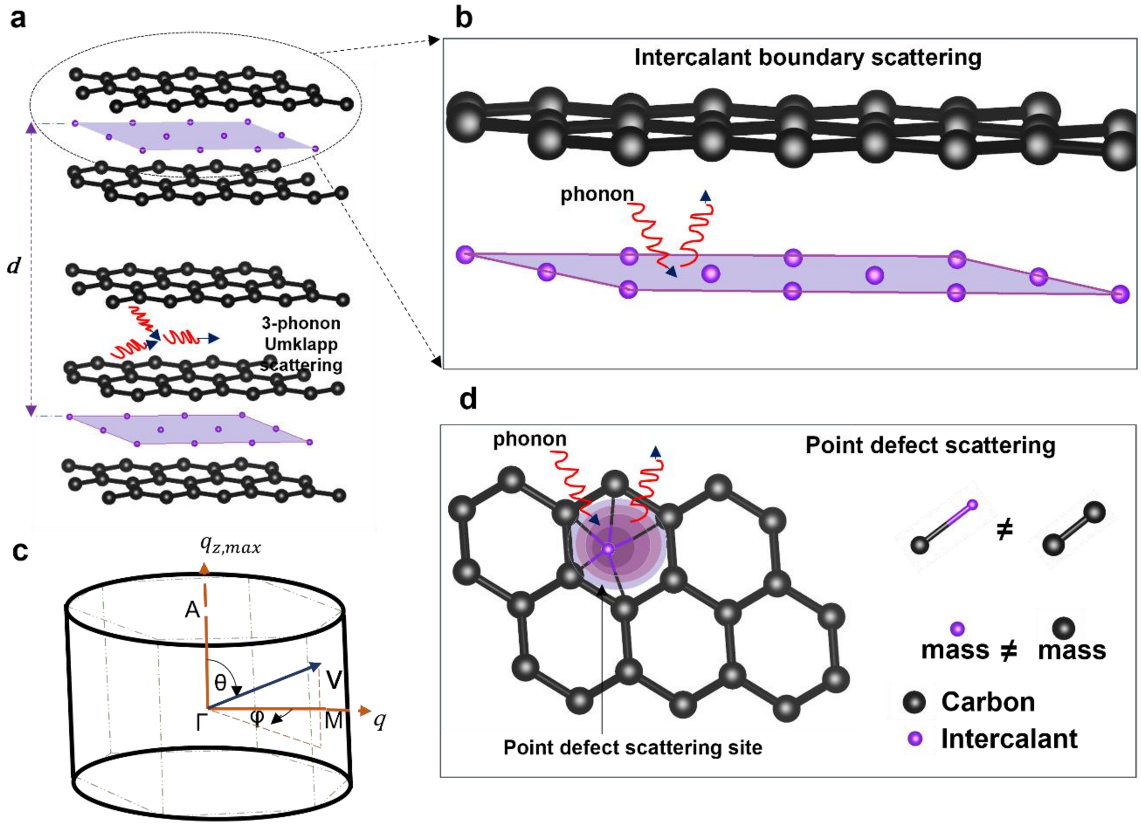

Figure S15. Thermal Transport Model. a, MLG with stage 3 intercalation. **b,** Phonon boundary scattering at the intercalant layer. **c,** Cylindrical approximation of hexagonal Brillouin zone with Γ, A and M symmetry points. **d,** Point defect scattering site due to the presence of intercalant.

In terms of scattering that limits the lattice thermal conductivity, we considered three phonon scattering processes in this model: Umklapp scattering τ_U^{-1}, boundary scattering τ_B^{-1}, and point defect scattering τ_{PD}^{-1}. Total phonon relaxation time τ is calculated by adding these scattering terms using Matthiessen's rule: $\tau^{-1} = \tau_U^{-1} + \tau_B^{-1} + \tau_{PD}^{-1}$. The Umklapp or three phonon scattering process defines limitation of thermal transport only due to anharmonicity in crystals, and is expressed as

$$\tau_U^{-1} = \frac{\gamma^2 k_B T \omega^2}{M v^2 \omega_{max}} \tag{S19}$$

where γ and ω_{max} are the Grüneisen parameters and maximum frequencies respectively for each phonon branch(*38*) The frequency, ω is described based on linear dispersion profile of the phonons. M is the mass of carbon atom and T is the temperature.

The boundary scattering term describes the limitation of mean free path of phonons at the MLG grain boundaries and cross plane boundary, expressed as,

$$\tau_B^{-1} = \frac{2\vec{v} \cdot \hat{n}_{\parallel}}{D_g} + \frac{2\vec{v} \cdot \hat{n}_{\perp}}{d_i} \tag{S20}$$

Here, D_g is the grain size along the in-plane direction, measured as 1.2±0.5 µm from SEM images. For non-intercalated state d_i is the thickness of the MLG sheet. However, for intercalated state of the device, we attributed the intercalation layers as the boundaries that limit mean free path of phonons along the cross-plane direction. In that scenario $d_i$is defined as the distance between intercalant layers. The XRD data used for determining intercalation stages, is utilised to establish a stepwise relationship between applied voltage and cross-plane boundary scattering distance d_i. The boundary scattering is assumed to be completely diffuse.

The point defect scattering term accounts the phonon scattering due to intrinsic impurities as well as the inclusion of intercalants. The defect scattering rate is defined as,

$$\tau_{Pd}^{-1} = \frac{2\pi\, \omega^3 \Gamma}{{\omega_{max}}^2} \tag{S21}$$

Γ is the scattering strength which is governed by the concentration of defects. The defect scattering strength only for the intrinsic impurities in MLG is determined by fitting the measured conductivity values of the non-intercalated MLG. In order to establish a voltage dependence in the intercalated MLG we defined a semi empirical relation with the previously measured sheet resistance as,

$$\frac{1}{R(V)} = \frac{1}{R_0} + b\Gamma(V) \tag{S22}$$

where b is a scaling parameter that is fitted for the measured thermal conductivity of the MLG at 0 V and 2.5 V. A parametric study was carried out to illustrate the comparative impact between the cross-plane directional boundary scattering and defect scattering. Thermal conductivity is limited by the boundary scattering than defect scattering as shown in Figure S16. Without the boundary scattering at the intercalate layers (d_i= 100 nm), even with very high defect scattering strength the in-plane conductivity fails to reach the measured value of intercalated MLG. On the other hand, with d_i

becoming 1 nm (cross-planar boundary scattering distance at stage 3 intercalation), thermal conductivity reaches the measured value having negligible defect scattering strength.

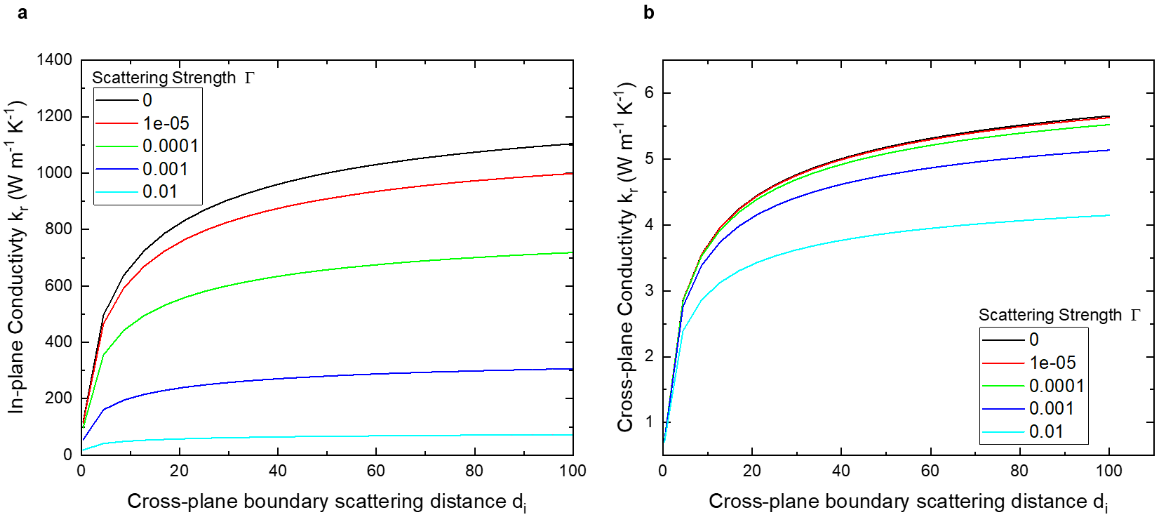

Figure S16. Thermal Transport Model. a, In-plane **b,** cross-plane conductivity profiles for cross-plane boundary scattering distance (in nm) with varying defect scattering strength.

8.1 Electronic Contribution to thermal conductivity

The electronic contribution to thermal conductivity is calculated using the Wiedemann–Franz law (17),

$$k_e = L\sigma T \qquad \text{(S23)}$$

Where

k_e= *electronic contribution to thermal conductivity.*

L = Lorentz number

$\sigma = \frac{1}{R_s t}$

T *= Temperature, K*

σ is the electrical conductivity calaculated using the measured sheet resistance, R_s and the thickness of the multilayer graphene device, t. The electronic contribution is found to be almost negligible for the non-intercalated device as illustrated in Figure S17. The lattice thermal conductivity from phonons dominates in the 0-2.5 V region. As the device is intercalated, phonon contribution is diminished, and electronic contribution becomes the dominant heat transport mechanism.

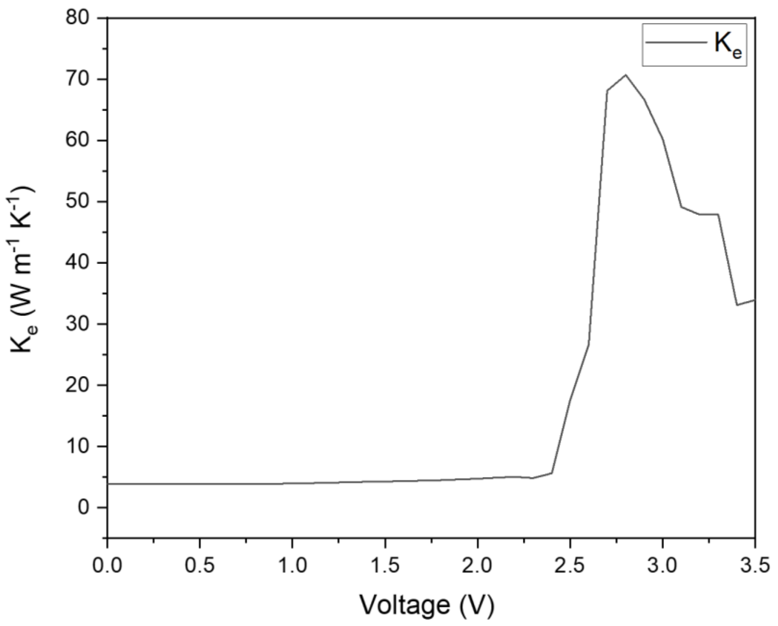

Figure S17. Electronic contribution. Calculated electronic thermal conductivity as a function of the intercalation device voltage.

8.2 Lithium intercalation Device to test electronic contribution:

We have tested the electronic contribution when the graphene layer is doped using Li intercalation which provides higher conductivity than the ionic liquid. WE have observed that on/off ratio is less than ionic liquid intercalation indicating higher electronic contribution. MTR data for Li intercalation device, displays a 6-fold modulation in the thermal conductivity.

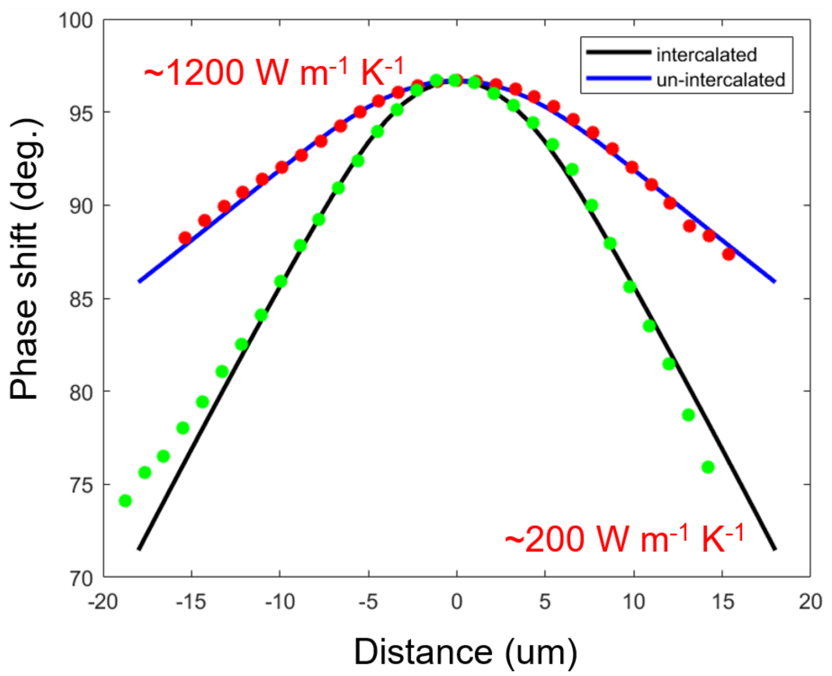

Figure S18. **Phase shift data acquired using MTR measurements of lithium intercalation device**. We observed modulation of in-plane thermal conductivity from ~1200 to ~200 W m^{-1} K^{-1}. Li intercalation provides a smaller on/off ratio, likely due to the large electronic contribution in the doped state.

8.3 Substrate effect on the device thermal conductivity:

The thermal conductivity of the multilayer graphene device is strongly affected by the substrate utilised. To minimise the substrate effect on the effective device thermal conductivity, we have utilised an ultra-thin polyethene substrate (Tab. S3). Additionally, when the polyethene substrate is

infused with the ionic liquid (IL) electrolyte the thermal conductivity diminished even further (Fig. S19), therefore we try to minimise the amount of IL used.

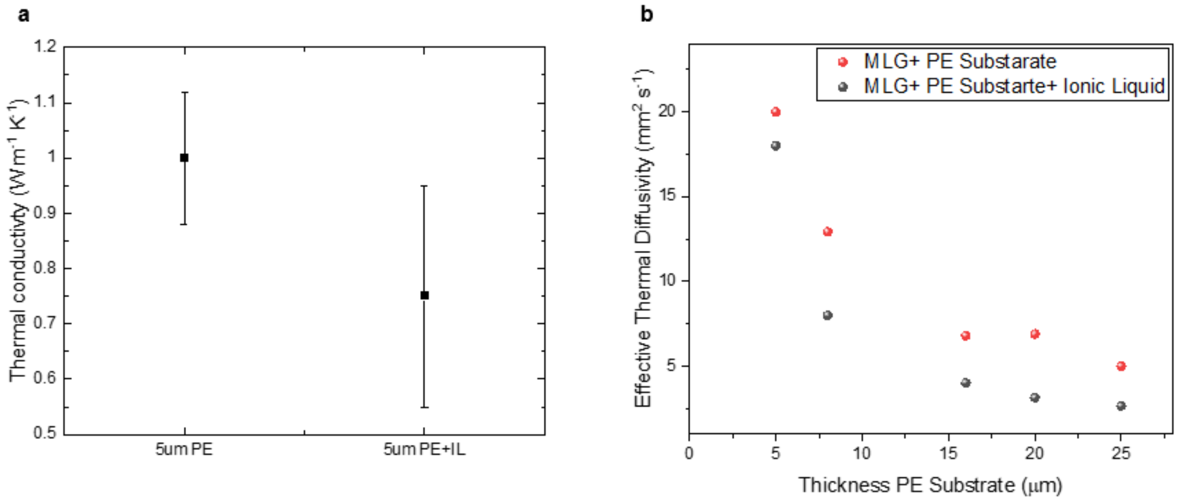

Figure S19. **Substrate effect on the device thermal conductivity. a,** Thermal conductivity of 5 micron thick polyethene with and without the ionic liquid electrolyte. **b,** Effective thermal diffusivity of the device as a function of the PE substrate thickness, without (Red) and with (Black) Ionic liquid electrolyte.

To prove that we are indeed approaching the intrinsic thermal conductivity modulation, we fabricated even thinner devices with 0.6um and 1um thick substrates. These new devices were measured using the IR thermogram. We observed a very consistent trend in the modulation vs substrate thickness. Using the IR thermogram, we obtained reversable modulation of 11 and 9.5-fold for 0.5 and 1 um substrate thickness. Figure S20 shows variation of the thermal conductivity for intercalated and de-intercalated samples as a function of substrate thickness. As the model expects, the device modulation is strongly dependent on the substrate thickness. As the substrate thickness is reduced, the thermal properties of the device approach MLG modulation measured with the MTR.

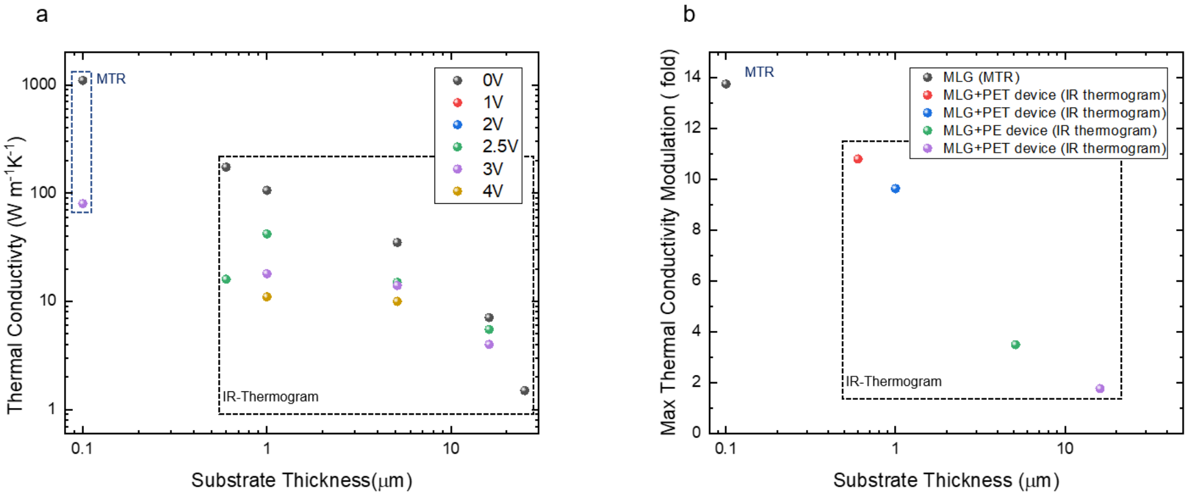

Figure S20. **Substrate effect on the device thermal conductivity. a.** thermal conductivity modulation as a function of the substrate thickness. **b.** thermal conductivity modulation (fold) as a function of the substrate thickness.

9. Scanning Thermal Microscopy

We implemented scanning thermal microscopy (SThM) and contact-mode atomic force microscopy with a special resistive AFM tip to measure the local thermal properties. At the apex of the tip, there is a resistive element which is heated up by passing a small current into it. When in contact with the sample, it dissipates its heat into the sample. A change in the tip temperature can provide useful information regarding the thermal properties of the specimen. It can be related to the specimen's local thermal conductivity variation. For example, when the tip is onto highly thermally conductive materials, the heat generated by the tip will be dissipated quicker, "cooling" the tip. When the tip is scanning a low thermally conductive material, the tip will heat up as less heat is dissipated.

Quantitative Understanding in Terms of Intercalation-Dependent Thermal Conductance Data: Our SThM data provides localized measurements of the thermal properties, whereas the thermoreflectance and IR thermograms present bulk thermal conductance data related to intercalation. Here's how they correlate:

Spatial Variability: SThM allowed us to identify potential areas or domains of varying thermal properties, indicative of heterogeneous intercalation or the presence of defects. Such regions would influence the bulk conductance measurements presented earlier.

Bulk vs. Local Correlation: Instances where the SThM data showed high thermal conductance coincided with regions of higher intercalation stages. Conversely, areas with low thermal conductance on the SThM correlated with efficiently doped areas with thinner layer numbers. We integrated the localized SThM measurements across the sample area to derive an averaged value. This average was found to be in good agreement with the bulk intercalation-dependent thermal conductance data, validating the consistency of our methodologies.

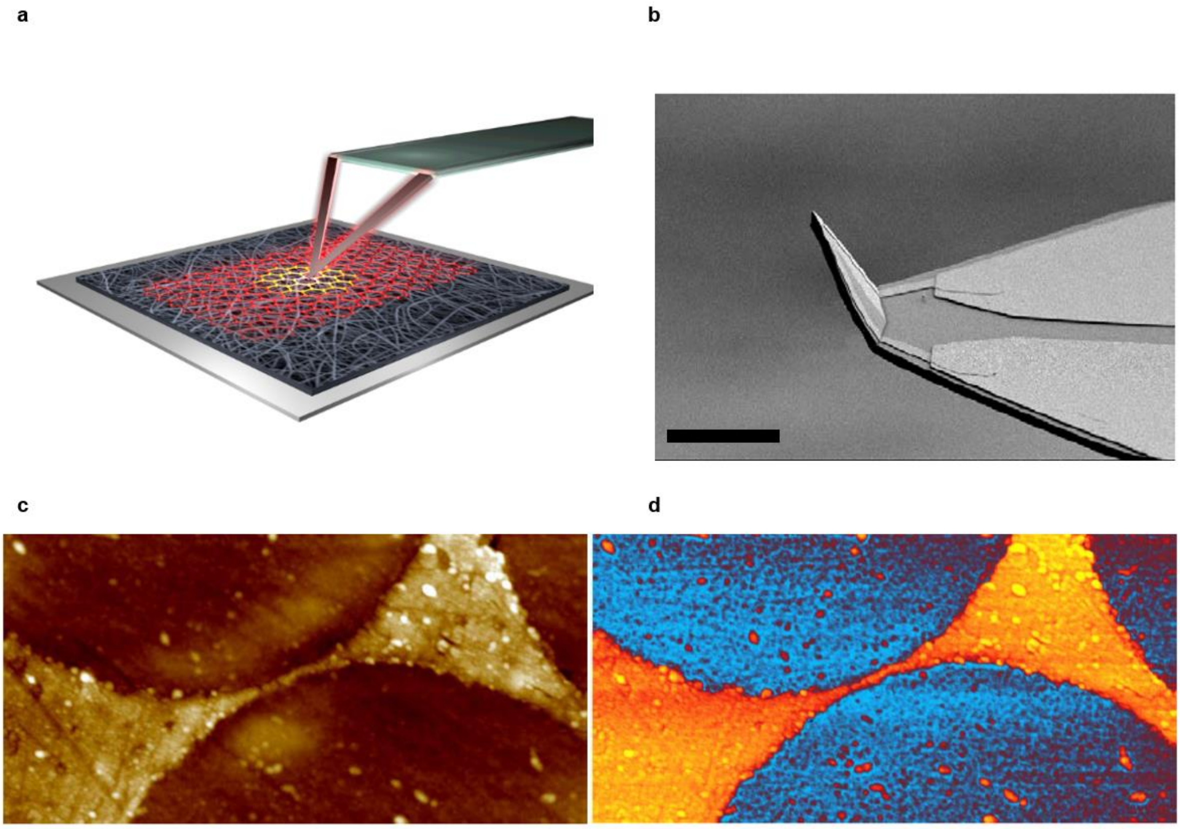

Figure S21. STHM technique. a. Schematic representing the working principle of the STHM technique. A resistive element is located at the apex of the tip, which heats up the tip when a current is applied. Measuring the change in temperature of the resistive tip can provide useful information regarding the thermal properties of the specimen. **b**. SEM picture of the STHM tip, scale bar 20 µm. **c, d.** Topography and STHM image of carbon fiber in epoxy matrix (image size 2 µm × 8 µm).

The SThM characterisation technique is qualitative as it is not independent of the morphology. For example, when the tip is mapping a wrinkle or void, the SThM signal is misled by the heat convection mechanism, which cools down the tip. The SThM required the fabrication of ultra-flat samples, which were produced as follows: a highly oriented pyrolytic graphite was exfoliated via micromechanical cleavage using plasma-treated Porous Polyethylene (PE). Subsequently, the PE membrane was soaked with ionic liquid electrolyte and deposited onto a glass substrate coated with 60 nm platinum. Finally, the graphite flakes were wired using silver paste. Applying a voltage between the graphite and counter platinum electrode enables the intercalation of the graphite. The MLG and graphite were grounded with a common ground to avoid any parasite current, which could interfere with the measurements. The change in the Sthm tip resistance is accurately measured by the Wheatstone bridge, which is balanced by utilising a variable resistor. Before each SThM

measurement, the tip resistance was matched with the variable resistor in the Wheatstone bridge to maximise its accuracy.

The thermal AFM data shows that intercalation is not a homogeneous process, which starts from the edges and sample defect. Thanks to Pauli blocking, the intercalation process can be observed in the IR thermogram (Fig. S22a) and optical microscope of Bruker Nano IR3 (Fig. S22b). Topography maps (Fig. S22c) provided useful information regarding the intercalation process. As expected, the graphite flakes expand as the intercalation process occurs, in agreement with the XRD measurements performed.

The thickness of the multilayer graphene influences the intercalation process greatly. When the thickness of the film increases, the intercalation becomes slower and less efficient. Choosing graphene film with large thickness variation enable to visualise the intercalation process.

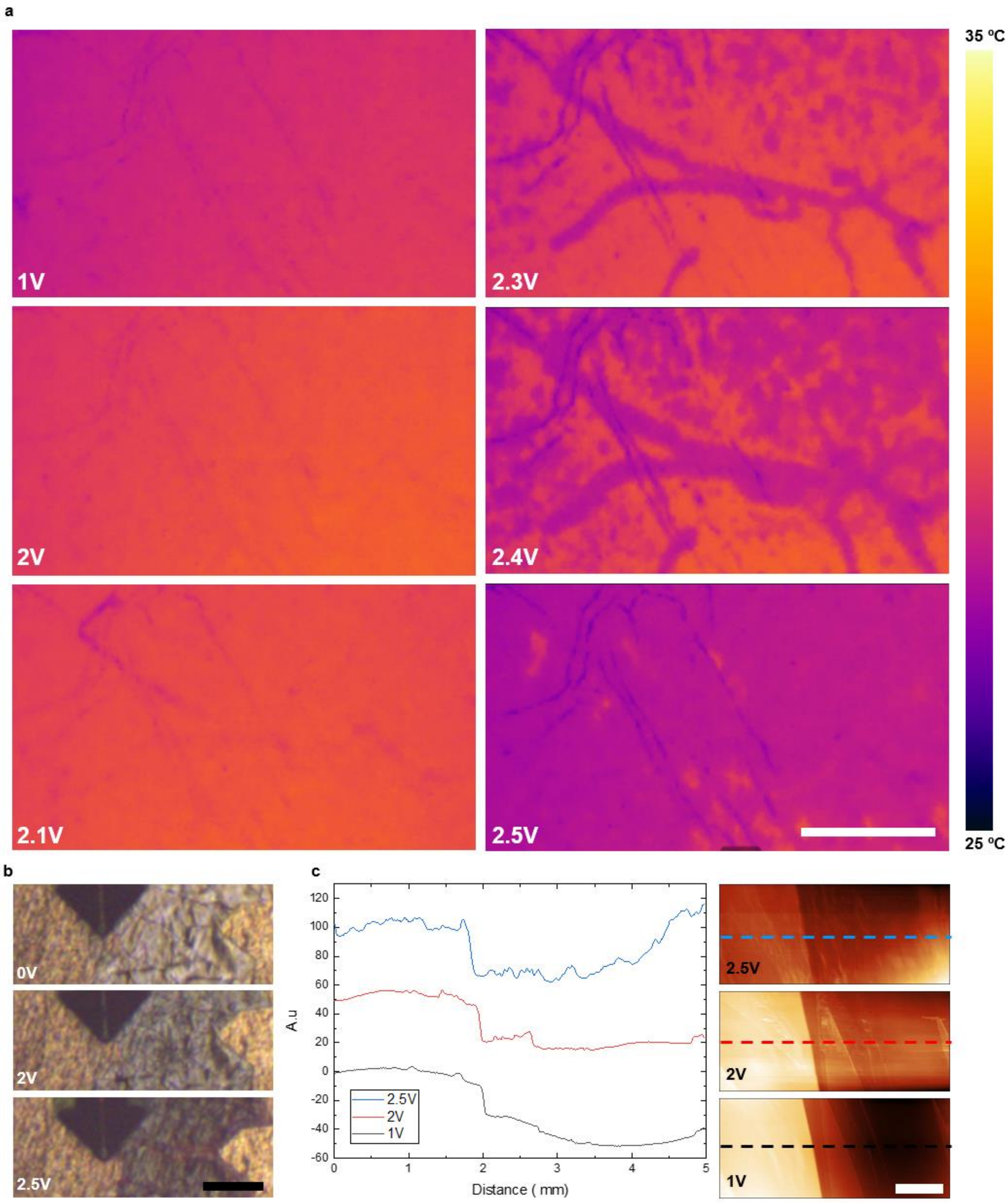

Figure S22. Scanning thermal microscopy. a, Thermogram images acquired at different voltages, highlighting the inhomogeneity of the intercalation process, which starts to form the defects such as edges and wrinkles (scale bar 2.5 mm). **b**, Nano IR3 optical microscope images acquired while scanning the samples at different voltages (scale bar 50 µm). The graphite flake beneath the tip gradually changes colours as the intercalation occurs due to Pauli blocking. **c**, height profile extracted from the topology map acquired at a different voltage. The graphite expands as the intercalation process occurs, procuring a change in the topology map (scale bar 1 µm).

10. **Further Discussion on Heat Steering:**

A new set of devices were fabricated to enhance further the heat flow control capabilities (Video S2). The devices follow the same layered structure as the previous devices with the difference of two bottom electrodes configured as in figure S23a.

The larger electrode is used to intercalate the MLG by applying a – ΔV. The smaller electrode is used to prevent the intercalation of MLG above the electrode (+ ΔV). A laser is used to heat up the centre of the device, and an infrared camera monitors the heat diffusion. Figure S23b represent three thermograms corresponding to the pristine device, intercalated device (the majority of the device is intercalated) and deintercalated device.

In the pristine case, the thermal diffusivity is uniform throughout the device, meaning that heat is radially diffusing. The thermal diffusivity becomes anisotropic when the device is intercalated. The intercalated area displays a thermal diffusivity which is halved compared to the un-intercalated channel (Fig. S23c). As a result, the channel will increase its temperature as more heat will preferentially flow through the channel. When the device is fully deintercalated, the heat flows radially once again, and no thermal diffusivity anisotropies are recorded.

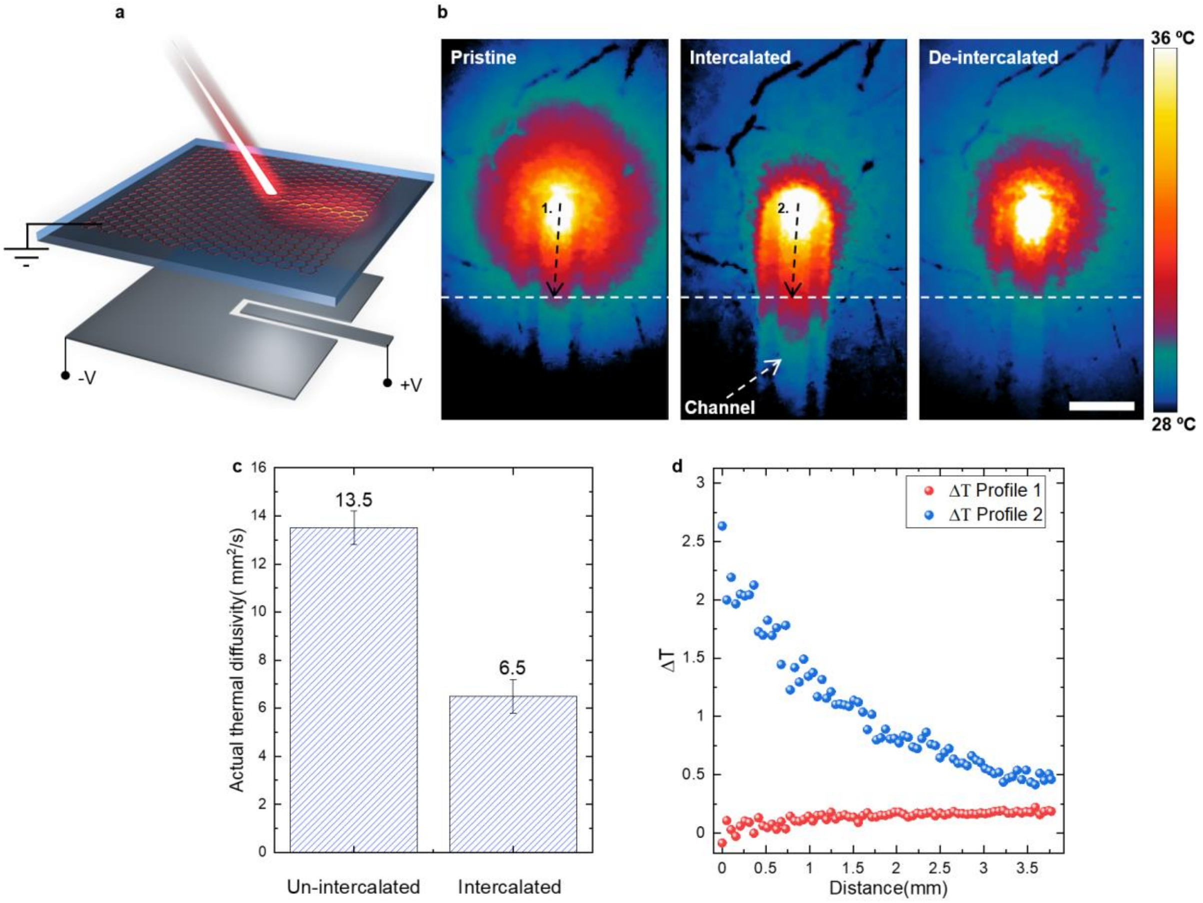

Figure S23. Heat Steering. a, Schematic of the device structures, including a top MLG layer, a separator and two bottom electrodes. A laser is used to heat the sample locally. **b,** Three thermograms were captured at three different applied voltage configurations (pristine, intercalated and un-intercalated). **c,** Thermal diffusivity for the intercalated and intercalated area. **d,** Temperature profile for the pristine and intercalated device. the graph highlights that the temperature in the un-intercalated channel increases when the device rest of the area intercalated suggesting that more heat is carried through the channel. Scale bar 1mm

11. Time response of the devices:

Both thermo-reflectance and IR thermogram measurements requires longer time (~1 mins acquisition time) than the switching time of the device (~1sec). Therefore, these methods are not suitable for the characterization of the time-dependent behavior of the thermal switch. To provide more insight we have characterized the time response from the infrared emissivity modulation which is directly related to the intercalation process thus the operation of the device. Figure S24 shows the infrared characterization of the device using a far-IR camera. The square waveform is applied to the device and the emissivity of the device is measured using a far-IR thermal camera. The device is placed on a hot plate at 40°C. The device consumes 15 mW/cm^2 peak power. Total energy stored in the device is estimated around 22 mJ/cm^2.

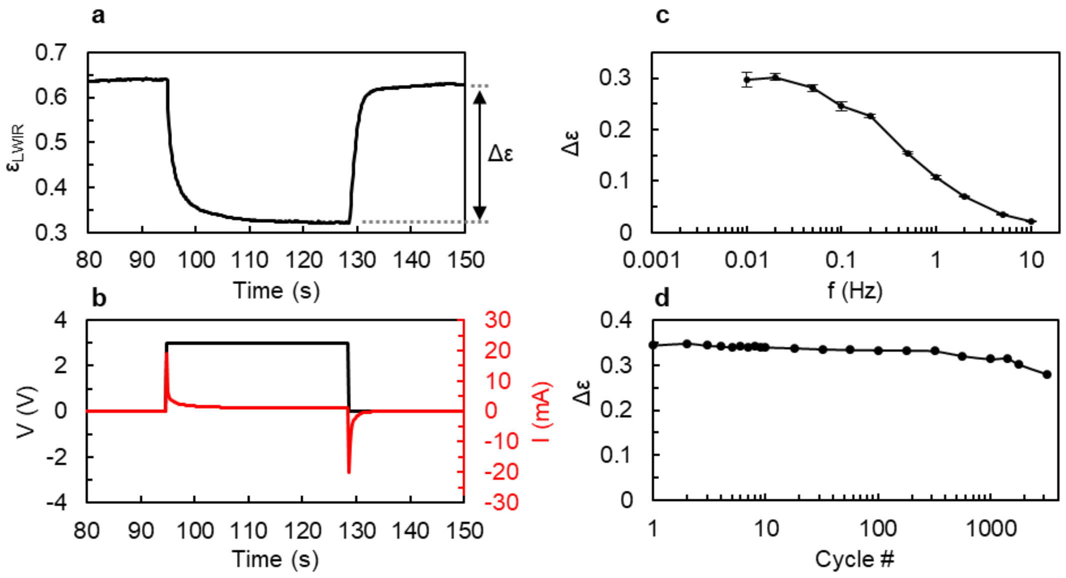

Figure S24. Time response of the devices. Time response of the devices: a, The variation of the emissivity of the device as a function of time. B, the voltage waveform and the transient current. C, shows the emissivity modulation as a function of frequency of the applied voltage. d, Long term stability of the emissivity modulation.

12: Testing the metallic transducer for MTR measurements:

We performed additional MTR measurements on MLG with the metal transducer, 45 nm Al coating (Fig S25). The results agree with the reported MTR measurements without metal transducer (Fig 2a). We can conclude that the addition of Al layer did not impact the measured values, and the error of the measurement is larger enough to don't appreciate any notable variation related to not having an Al coating.

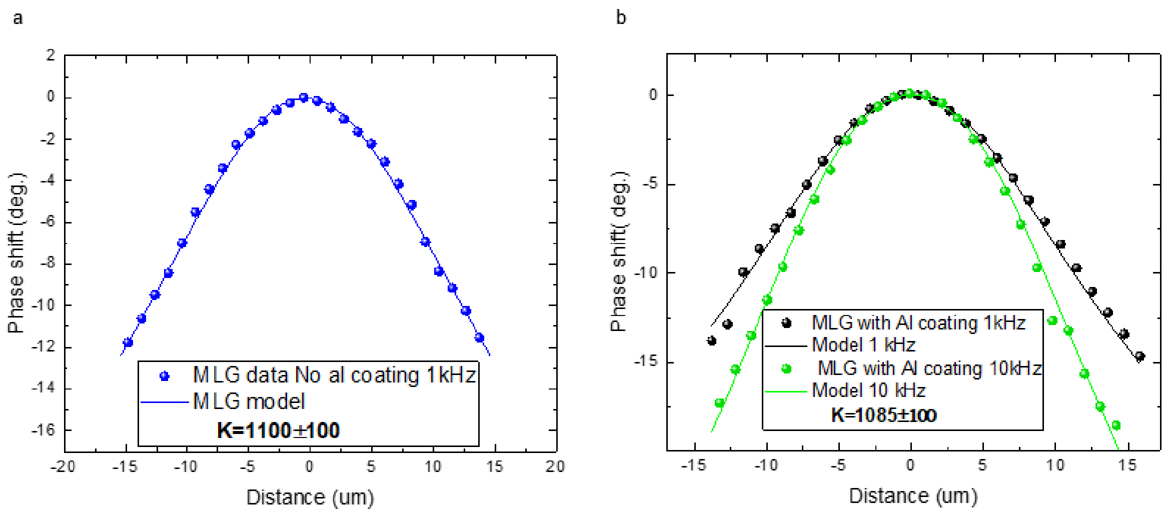

Figure S25. Testing the metallic transducer. MTR phase shift data for MLG (100 nm) coated without and with 45 nm Al metal transducer. the calculated thermal conductivities are in well agreement. Without metal transducer K=1100±100 m^{-1} K^{-1} and with metal transducer K=1085±100 m^{-1} K^{-1}.

REFERENCES AND NOTES

1. G. Wehmeyer, T. Yabuki, C. Monachon, J. Wu, C. Dames, Thermal diodes, regulators, and switches: Physical mechanisms and potential applications. *Appl. Phys. Rev.* **4**, 041304 (2017).

2. D. G. Cahill, W. K. Ford, K. E. Goodson, G. D. Mahan, A. Majumdar, H. J. Maris, R. Merlin, S. R. Phillpot, Nanoscale thermal transport. *J. Appl. Phys.* **93**, 793–818 (2003).

3. E. S. Toberer, L. L. Baranowski, C. Dames, Advances in thermal conductivity. *Annu. Rev. Mat. Res.* **42**, 179–209 (2012).

4. T. Swanson, "NASA's new thermal management systems roadmap; what's in it, what it means," paper presented at the Aerospace Thermal Control Workshop, El Segundo, CA, 22 March 2016.

5. S. Cui, F. Jiang, N. Song, L. Shi, P. Ding, Flexible films for smart thermal management: Influence of structure construction of a two-dimensional graphene network on active heat dissipation response behavior. *ACS Appl. Mater. Interfaces* **11**, 30352–30359 (2019).

6. J. Hansson, T. M. J. Nilsson, L. Ye, J. Liu, Novel nanostructured thermal interface materials: A review. *Int. Mater. Rev.* **63**, 22–45 (2018).

7. M. Li, H. Wu, E. M. Avery, Z. Qin, D. P. Goronzy, H. D. Nguyen, T. Liu, P. S. Weiss, Y. Hu, Electrically gated molecular thermal switch. *Science* **382**, 585–589 (2023).

8. A. A. Balandin, Thermal properties of graphene and nanostructured carbon materials. *Nat. Mater.* **10**, 569–581 (2011).

9. S. Yiğen, A. R. Champagne, Wiedemann–Franz relation and thermal-transistor effect in suspended graphene. *Nano Lett.* **14**, 289–293 (2014).

10. C. Liu, Y. Si, H. Zhang, C. Wu, S. Deng, Y. Dong, Y. Li, M. Zhuo, N. Fan, B. Xu, P. Lu, L. Zhang, X. Lin, X. Liu, J. Yang, Z. Luo, S. Das, L. Bellaiche, Y. Chen, Z. Chen, Low voltage–driven high-performance thermal switching in antiferroelectric $PbZrO_3$ thin films. *Science* **382**, 1265–1269 (2023).

11. Q. Lu, S. Huberman, H. Zhang, Q. Song, J. Wang, G. Vardar, A. Hunt, I. Waluyo, G. Chen, B. Yildiz, Bi-directional tuning of thermal transport in SrCoO*x* with electrochemically induced phase transitions. *Nat. Mater.* **19**, 655–662 (2020).

12. G. Zhu, J. Liu, Q. Zheng, R. Zhang, D. Li, D. Banerjee, D. G. Cahill, Tuning thermal conductivity in molybdenum disulfide by electrochemical intercalation. *Nat. Commun.* **7**, 13211 (2016).

13. Y. Xiong, N. C. Lai, Y. C. Lu, D. Xu, Tuning thermal conductivity of bismuth selenide nanoribbons by reversible copper intercalation. *Int. J. Heat Mass Transf.* **159**, 120077 (2020).

14. M. E. Chen, M. M. Rojo, F. Lian, J. Koeln, A. Sood, S. M. Bohaichuk, C. M. Neumann, S. G. Garrow, K. E. Goodson, A. G. Alleyne, E. Pop, Graphene-based electromechanical thermal switches. *2D Mater.* **8**, 035055 (2021).

15. J. A. Tomko, A. Pena-Francesch, H. Jung, M. Tyagi, B. D. Allen, M. C. Demirel, P. E. Hopkins, Tunable thermal transport and reversible thermal conductivity switching in topologically networked bio-inspired materials. *Nat. Nanotechnol.* **13**, 959–964 (2018).

16. T. Swoboda, K. Klinar, A. S. Yalamarthy, A. Kitanovski, M. Muñoz Rojo, Solid-state thermal control devices. *Adv. Electron. Mater.* **7**, 2000625 (2021).

17. J. P. Issi, J. Heremans, M. S. Dresselhaus, Electronic and lattice contributions to the thermal conductivity of graphite intercalation compounds. *Phys. Rev. B* **27**, 1333–1347 (1983).

18. R. Matsumoto, Y. Hoshina, N. Akuzawa, Thermoelectric properties and electrical transport of graphite intercalation compounds. *Mater. Trans.* **50**, 1607–1611 (2009).

19. J. S. Kang, M. Ke, Y. Hu, Ionic intercalation in two-dimensional van der Waals Materials: In situ characterization and electrochemical control of the anisotropic thermal conductivity of black phosphorus. *Nano Lett.* **17**, 1431–1438 (2017).

20. A. Sood, F. Xiong, S. Chen, H. Wang, D. Selli, J. Zhang, C. J. McClellan, J. Sun, D. Donadio, Y. Cui, E. Pop, K. E. Goodson, An electrochemical thermal transistor. *Nat. Commun.* **9**, 4510 (2018).

21. J. Cho, M. D. Losego, H. G. Zhang, H. Kim, J. Zuo, I. Petrov, D. G. Cahill, P. V. Braun, Electrochemically tunable thermal conductivity of lithium cobalt oxide. *Nat. Commun.* **5**, 4035 (2014).

22. Z. Bian, Q. Yang, M. Yoshimura, H. J. Cho, J. Lee, H. Jeen, T. Endo, Y. Matsuo, H. Ohta, Solid-state electrochemical thermal transistors with strontium cobaltite-strontium ferrite solid solutions as the active layers. *ACS Appl. Mater. Interfaces* **15**, 23512–23517 (2023).

23. Q. Yang, H. J. Cho, Z. Bian, M. Yoshimura, J. Lee, H. Jeen, J. Lin, J. Wei, B. Feng, Y. Ikuhara, H. Ohta, Solid-state electrochemical thermal transistors. *Adv. Funct. Mater.* **33**, 2214939 (2023).

24. A. Block, A. Principi, N. C. H. Hesp, A. W. Cummings, M. Liebel, K. Watanabe, T. Taniguchi, S. Roche, F. H. L. Koppens, N. F. van Hulst, K.-J. Tielrooij, Observation of giant and tunable thermal diffusivity of a Dirac fluid at room temperature. *Nat. Nanotechnol.* **16**, 1195–1200 (2021).

25. J. Shin, S. Kim, H. Park, H. Won Jang, D. G. Cahill, P. V. Braun, Thermal conductivity of intercalation, conversion, and alloying lithium-ion battery electrode materials as function of their state of charge. *Curr. Opin. Solid State Mater. Sci.* **26**, 100980 (2022).

26. Y. Zeng, D. Chalise, Y. Fu, J. Schaadt, S. Kaur, V. Battaglia, S. D. Lubner, R. S. Prasher, *Operando* spatial mapping of lithium concentration using thermal-wave sensing. *Joule* **5**, 2195–2210 (2021).

27. Y. Machida, N. Matsumoto, T. Isono, K. Behnia, Phonon hydrodynamics and ultrahigh-room-temperature thermal conductivity in thin graphite. *Science* **367**, 309–312 (2020).

28. O. Salihoglu, H. B. Uzlu, O. Yakar, S. Aas, O. Balci, N. Kakenov, S. Balci, S. Olcum, S. Süzer, C. Kocabas, Graphene-based adaptive thermal camouflage. *Nano Lett.* **18**, 4541–4548 (2018).

29. M. S. Ergoktas, G. Bakan, P. Steiner, C. Bartlam, Y. Malevich, E. Ozden-Yenigun, G. He, N. Karim, P. Cataldi, M. A. Bissett, I. A. Kinloch, K. S. Novoselov, C. Kocabas, Graphene-enabled adaptive infrared textiles. *Nano Lett.* **20**, 5346–5352 (2020).

30. M. Pawlak, M. Streza, C. Morari, K. Strzałkowski, M. Depriester, M. Chirtoc, Quantitative thermal wave phase imaging of an IR semi-transparent GaAs wafer using IR lock-in thermography. *Meas. Sci. Technol.* **28**, 025008 (2017).

31. M. F. Riyad, V. Chauhan, M. Khafizov, Implementation of a multilayer model for measurement of thermal conductivity in ion beam irradiated samples using a modulated thermoreflectance approach. *J. Nucl. Mater.* **509**, 134–144 (2018).

32. V. S. Chauhan, A. Abdullaev, Z. Utegulov, J. O'Connell, V. Skuratov, M. Khafizov, Simultaneous characterization of cross- and in-plane thermal transport in insulator patterned by directionally aligned nano-channels. *AIP Adv.* **10**, 015304 (2020).

33. Y. Wang, D. H. Hurley, E. P. Luther, M. F. Beaux, D. R. Vodnik, R. J. Peterson, B. L. Bennett, I. O. Usov, P. Yuan, X. Wang, M. Khafizov, Characterization of ultralow thermal conductivity in anisotropic pyrolytic carbon coating for thermal management applications. *Carbon* **129**, 476–485 (2018).

34. M. S. Ergoktas, G. Bakan, E. Kovalska, L. W. le Fevre, R. P. Fields, P. Steiner, X. Yu, O. Salihoglu, S. Balci, V. I. Fal'ko, K. S. Novoselov, R. A. W. Dryfe, C. Kocabas, Multispectral graphene-based electro-optical surfaces with reversible tunability from visible to microwave wavelengths. *Nat. Photonics* **15**, 493–498 (2021).

35. X. Zhang, N. Sukpirom, M. M. Lerner, Graphite intercalation of bis (trifluoromethanesulfonyl) imide and other anions with perfluoroalkanesulfonyl substituents. *Mater. Res. Bull.* **34**, 363–372 (1999).

36. G. Schmuelling, T. Placke, R. Kloepsch, O. Fromm, H.-W. Meyer, S. Passerini, M. Winter, X-ray diffraction studies of the electrochemical intercalation of bis (trifluoromethanesulfonyl) imide anions into graphite for dual-ion cells. *J. Power Sources* **239**, 563–571 (2013).

37. P. G. Klemens, D. F. Pedraza, Thermal conductivity of graphite in the basal plane. *Carbon* **32**, 735–741 (1994).

38. D. L. Nika, A. S. Askerov, A. A. Balandin, Anomalous size dependence of the thermal conductivity of graphene ribbons. *Nano Lett.* **12**, 3238–3244 (2012).

39. C. A. Dennett, W. R. Deskins, M. Khafizov, Z. Hua, A. Khanolkar, K. Bawane, L. Fu, J. M. Mann, C. A. Marianetti, L. He, D. H. Hurley, A. El-Azab, An integrated experimental and computational investigation of defect and microstructural effects on thermal transport in thorium dioxide. *Acta Mater.* **213**, 116934 (2021).

40. X. Qian, X. Gu, M. S. Dresselhaus, R. Yang, Anisotropic tuning of graphite thermal conductivity by lithium intercalation. *J. Phys. Chem. Lett.* **7**, 4744–4750 (2016).

41. E. Puyoo, S. Grauby, J.-M. Rampnoux, E. Rouvière, S. Dilhaire, Scanning thermal microscopy of individual silicon nanowires. *J. Appl. Phys.* **109**, 024302 (2011).

42. W. Bao, J. Wan, X. Han, X. Cai, H. Zhu, D. Kim, D. Ma, Y. Xu, J. N. Munday, H. D. Drew, M. S. Fuhrer, L. Hu, Approaching the limits of transparency and conductivity in graphitic materials through lithium intercalation. *Nat. Commun.* **5**, 4224 (2014).

43. C. Wan, X. Gu, F. Dang, T. Itoh, Y. Wang, H. Sasaki, M. Kondo, K. Koga, K. Yabuki, G. J. Snyder, R. Yang, K. Koumoto, Flexible n-type thermoelectric materials by organic intercalation of layered transition metal dichalcogenide TiS_2. *Nat. Mater.* **14**, 622–627 (2015).

44. S. Dhara, H. S. Solanki, A. P. R, V. Singh, S. Sengupta, B. A. Chalke, A. Dhar, M. Gokhale, A. Bhattacharya, M. M. Deshmukh, Tunable thermal conductivity in defect engineered nanowires at low temperatures. *Phys. Rev. B* **84**, 121307 (2011).

45. B. M. Foley, M. Wallace, J. T. Gaskins, E. A. Paisley, R. L. Johnson-Wilke, J.-W. Kim, P. J. Ryan, S. Trolier-McKinstry, P. E. Hopkins, J. F. Ihlefeld, Voltage-controlled bistable thermal conductivity in suspended ferroelectric thin-film membranes. *ACS Appl. Mater. Interfaces* **10**, 25493–25501 (2018).

46. J. F. Ihlefeld, B. M. Foley, D. A. Scrymgeour, J. R. Michael, B. B. McKenzie, D. L. Medlin, M. Wallace, S. Trolier-McKinstry, P. E. Hopkins, Room-temperature voltage tunable phonon thermal conductivity via reconfigurable interfaces in ferroelectric thin films. *Nano Lett.* **15**, 1791–1795 (2015).

47. J. Crossno, J. K. Shi, K. Wang, X. Liu, A. Harzheim, A. Lucas, S. Sachdev, P. Kim, T. Taniguchi, K. Watanabe, T. A. Ohki, K. C. Fong, Observation of the Dirac fluid and the breakdown of the Wiedemann-Franz law in graphene. *Science* **351**, 1058–1061 (2016).

48. S. Deng, J. Yuan, Y. Lin, X. Yu, D. Ma, Y. Huang, R. Ji, G. Zhang, N. Yang, Electric-field-induced modulation of thermal conductivity in poly (vinylidene fluoride). *Nano Energy* **82**, 105749 (2021).

49. X. Zhao, J. C. Wu, Z. Y. Zhao, Z. Z. He, J. D. Song, J. Y. Zhao, X. G. Liu, X. F. Sun, X. G. Li, Heat switch effect in an antiferromagnetic insulator $Co_3V_2O_8$. *Appl. Phys. Lett.* **108**, 242405 (2016).

50. X. M. Wang, C. Fan, Z. Y. Zhao, W. Tao, X. G. Liu, W. P. Ke, X. Zhao, X. F. Sun, Large magnetothermal conductivity of $HoMnO_3$ single crystals and its relation to the magnetic-field-induced transitions of magnetic structure. *Phys. Rev. B* **82**, 094405 (2010).

51. H.-T. Huang, M.-F. Lai, Y.-F. Hou, Z.-H. Wei, Influence of magnetic domain walls and magnetic field on the thermal conductivity of magnetic nanowires. *Nano Lett.* **15**, 2773–2779 (2015).

52. J. Kimling, R. B. Wilson, K. Rott, J. Kimling, G. Reiss, D. G. Cahill, Spin-dependent thermal transport perpendicular to the planes of Co/Cu multilayers. *Phys. Rev. B* **91**, 144405 (2015).

53. J. Shin, M. Kang, T. Tsai, C. Leal, P. V. Braun, D. G. Cahill, Thermally functional liquid crystal networks by magnetic field driven molecular orientation. *ACS Macro Lett.* **5**, 955–960 (2016).

54. S. Lee, K. Hippalgaonkar, F. Yang, J. Hong, C. Ko, J. Suh, K. Liu, K. Wang, J. J. Urban, X. Zhang, C. Dames, S. A. Hartnoll, O. Delaire, J. Wu, Anomalously low electronic thermal conductivity in metallic vanadium dioxide. *Science* **355**, 371–374 (2017).

55. R. Shrestha, Y. Luan, S. Shin, T. Zhang, X. Luo, J. S. Lundh, W. Gong, M. R. Bockstaller, S. Choi, T. Luo, R. Chen, K. Hippalgaonkar, S. Shen, High-contrast and reversible polymer thermal regulator by structural phase transition. *Sci. Adv.* **5**, eaax3777 (2019).

56. R. Nakayama, T. Takeuchi, Thermal rectification in bulk material through unusual behavior of electron thermal conductivity of Al-Cu-Fe icosahedral quasicrystal. *J. Electron. Mater.* **44**, 356–361 (2015).

57. J. Lee, E. Bozorg-Grayeli, S. Kim, M. Asheghi, H.-S. Philip Wong, K. E. Goodson, Phonon and electron transport through $Ge_2Sb_2Te_5$ films and interfaces bounded by metals. *Appl. Phys. Lett.* **102**, 191911 (2013).

58. S. D. Lubner, J. Choi, G. Wehmeyer, B. Waag, V. Mishra, H. Natesan, J. C. Bischof, C. Dames, Reusable bi-directional 3ω sensor to measure thermal conductivity of 100- μm thick biological tissues. *Rev. Sci. Instrum.* **86**, 014905 (2015).

59. K. Dahal, Q. Zhang, Y. Wang, I. K. Mishra, Z. Ren, V–VO_2 core–shell structure for potential thermal switching. *RSC Adv.* **7**, 33775–33781 (2017).

60. R. Zheng, J. Gao, J. Wang, G. Chen, Reversible temperature regulation of electrical and thermal conductivity using liquid–solid phase transitions. *Nat. Commun.* **2**, 289 (2011).

61. P. C. Sun, Y. L. Wu, J. W. Gao, G. A. Cheng, G. Chen, R. T. Zheng, Room temperature electrical and thermal switching CNT/hexadecane composites. *Adv. Mater.* **25**, 4938–4943 (2013).

62. D.-W. Oh, C. Ko, S. Ramanathan, D. G. Cahill, Thermal conductivity and dynamic heat capacity across the metal-insulator transition in thin film VO_2. *Appl. Phys. Lett.* **96**, 151906 (2010).

63. Q. Zheng, G. Zhu, Z. Diao, D. Banerjee, D. G. Cahill, High contrast thermal conductivity change in Ni–Mn–In Heusler alloys near room temperature. *Adv. Eng. Mater.* **21**, 1801342 (2019).

64. J. J. Martínez-Flores, L. Licea-Jimenez, S. A. Perez Garcia, J. Alvarez-Quintana, Magnon-mediated thermal rectification with forward-bias and breakdown temperatures. *J. Appl. Phys.* **114**, 104904 (2013).

65. Q. Zheng, S. E. Murray, Z. Diao, A. Bhutani, D. P. Shoemaker, D. G. Cahill, Thermal transport through the magnetic martensitic transition in Mn_xM Ge (M = Co, Ni). *Phys. Rev. Mater.* **2**, 075401 (2018).

66. C. Li, Y. Ma, Z. Tian, Thermal switching of thermoresponsive polymer aqueous solutions. *ACS Macro Lett.* **7**, 53–58 (2018).

67. T. Du, Z. Xiong, L. Delgado, W. Liao, J. Peoples, R. Kantharaj, P. R. Chowdhury, A. Marconnet, X. Ruan, Wide range continuously tunable and fast thermal switching based on compressible graphene composite foams. *Nat. Commun.* **12**, 4915 (2021).

68. R. Xie, C. T. Bui, B. Varghese, Q. Zhang, C. H. Sow, B. Li, J. T. L. Thong, An electrically tuned solid-state thermal memory based on metal-insulator transition of single-crystalline VO_2 nanobeams. *Adv. Funct. Mater.* **21**, 1602–1607 (2011).

69. A. V. Talyzin, O. Andersson, B. Sundqvist, A. Kurnosov, L. Dubrovinsky, High-pressure phase transition in $LiBH_4$. *J. Solid State Chem.* **180**, 510–517 (2007).

70. J. Shin, J. Sung, M. Kang, X. Xie, B. Lee, K. M. Lee, T. J. White, C. Leal, N. R. Sottos, P. V. Braun, D. G. Cahill, Light-triggered thermal conductivity switching in azobenzene polymers. *Proc. Natl. Acad. Sci. U.S.A.* **116**, 5973–5978 (2019).

71. C. McGuire, K. Sawchuk, A. Kavner, Measurements of thermal conductivity across the B1-B2 phase transition in NaCl. *J. Appl. Phys.* **124**, 115902 (2018).

72. Y. Zeng, C.-L. Lo, S. Zhang, Z. Chen, A. Marconnet, Dynamically tunable thermal transport in polycrystalline graphene by strain engineering. *Carbon* **158**, 63–68 (2020).

73. Y. Gao, Q. Liu, B. Xu, Lattice mismatch dominant yet mechanically tunable thermal conductivity in bilayer heterostructures. *ACS Nano* **10**, 5431–5439 (2016).

74. C. Liu, V. Mishra, Y. Chen, C. Dames, Large thermal conductivity switch ratio in barium titanate under electric field through first-principles calculation. *Adv. Theory Simul.* **1**, 1800098 (2018).

75. X. Liu, G. Zhang, Y.-W. Zhang, Graphene-based thermal modulators. *Nano Res.* **8**, 2755–2762 (2015).

76. J. A. Seijas-Bellido, C. Escorihuela-Sayalero, M. Royo, M. P. Ljungberg, J. C. Wojdeł, J. Íñiguez, R. Rurali, A phononic switch based on ferroelectric domain walls. *Phys. Rev. B* **96**, 140101 (2017).

77. Y. Zhu, Heat-loss modified Angstrom method for simultaneous measurements of thermal diffusivity and conductivity of graphite sheets: The origins of heat loss in Angstrom method. *Int. J. Heat Mass Transf.* **92**, 784–791 (2016).

78. J. Zhang, E. M. Levenson-Falk, B. J. Ramshaw, D. A. Bonn, R. Liang, W. N. Hardy, S. A. Hartnoll, A. Kapitulnik, Anomalous thermal diffusivity in underdoped $YBa_2Cu_3O_{6+x}$. *Proc. Natl. Acad. Sci. U.S.A.* **114**, 5378–5383 (2017).

79. A. Feldman, Algorithm for solutions of the thermal diffusion equation in a stratified medium with a modulated heating source. *High Temp. High Press.* **31**, 293–298 (1999).

80. E. Solfiti, F. Berto, A review on thermophysical properties of flexible graphite. *Procedia Struct. Integr.* **26**, 187–198 (2020).

81. A. J. Williams, D. T. Burns, M. R. McEwen, "Measurement of the specific heat capacity of the electron-beam graphite calorimeter. NPL Report RSA (EXT)" (National Physical Laboratory, 1993), vol. 40, pp. 1–17.

82. S. Picard, D. T. Burns, P. Roger, Determination of the specific heat capacity of a graphite sample using absolute and differential methods. *Metrologia* **44**, 294–302 (2007).
